# Alterations of Urinary Microbiota in Type 2 Diabetes Mellitus with Hypertension and/or Hyperlipidemia

**DOI:** 10.3389/fphys.2017.00126

**Published:** 2017-03-03

**Authors:** Fengping Liu, Zongxin Ling, Yonghong Xiao, Qing Yang, Baohong Wang, Li Zheng, Ping Jiang, Lanjuan Li, Wei Wang

**Affiliations:** ^1^State Key Laboratory for Diagnosis and Treatment of Infectious Diseases, First Affiliated Hospital, School of Medicine, Zhejiang UniversityHangzhou, China; ^2^Nursing School, Yancheng Medical CollegeYancheng, China; ^3^Collaborative Innovation Center for Diagnosis and Treatment of Infectious Diseases, First Affiliated Hospital, School of Medicine, Zhejiang UniversityHangzhou, China; ^4^Department of Urology, First Affiliated Hospital, School of Medicine, Zhejiang UniversityHangzhou, China

**Keywords:** type 2 diabetes mellitus, hypertension, hyperlipidemia, *Lactobacillus*, Proteobacteria, urinary microbiota

## Abstract

Evidence shows urine specimens from different women have different populations of bacteria. The co-occurrence of hypertension and hyperlipidemia in those with diabetes may alter the composition of urine and the microenviroment of the bladder in which bacteria live. The aim of this study was to characterize the urinary microbiota in women with type 2 diabetes mellitus only and those with diabetes plus hypertension and/or hyperlipidemia, and to explore whether the composition of the urinary microbiota is affected by fasting blood glucose, blood pressure, and blood lipids. We enrolled 28 individuals with diabetes only, 24 with diabetes plus hypertension, 7 with diabetes plus hyperlipidemia, and 11 with diabetes plus both hypertension and hyperlipidemia. Modified midstream urine collection technique was designed to obtain urine specimens. Bacterial genomic DNA was isolated using magnetic beads and the urinary microbiota was analyzed using the Illumina MiSeq Sequencing System based on the V3-V4 hypervariable regions of the 16S rRNA gene. Among the four cohorts, the diabetes plus hypertension cohort had the highest relative abundance of Proteobacteria. In contrast, the diabetes plus hyperlipidemia cohort had the lowest relative abundance of Proteobacteria. In addition, *Escherichia* and *Gardnerella* were not found in the diabetes plus hyperlipidemia cohort but they were found in all of the other cohorts. *Cetobacterium* was only present in the diabetes plus hypertension cohort. The most abundant bacteria in the diabetes only and diabetes plus hyperlipidemia cohorts was *Lactobacillus*, while *Prevotella* was the most abundant bacteria in the diabetes plus hypertension and diabetes plus hypertension and hyperlipidemia cohorts. Moreover, the relative abundance of *Lactobacillus* was significantly lower in the diabetes plus hypertension cohort than in the diabetes only and diabetes plus hyperlipidemia cohorts. Several bacteria were correlated with the participants' fasting blood glucose, blood pressure, and blood lipids. In conclusion, hypertension and/or hyperlipidemia and other patient factors can affect the composition of the urinary microbiota in those with diabetes. The insights from this study could be used to develop microbiota-based treatment for comorbid conditions, including urinary tract infections, in those with diabetes.

## Introduction

The number of people around the world with diabetes has risen from 108 million in 1980 to 422 million in 2014 (World Health Organization, 2016). Type 2 diabetic mellitus (T2DM) accounts for approximately 90–95% of cases of diabetes (Rubino, 2008). Up to 75% of the adult population with diabetes has hypertension and approximately 52.8% of those with diabetes have hyperlipidemia (de Sereday et al., 2004; Long and Dagogo-Jack, 2011).

Recent evidence has shown that urinary microbiota profiles are complex and they vary considerably between individuals, both in women and men (Dong et al., 2011; Siddiqui et al., 2011, 2012; Fouts et al., 2012; Wolfe et al., 2012; Lewis et al., 2013; Fricke et al., 2014; Nienhouse et al., 2014; Pearce et al., 2014, 2015; Santiago-Rodriguez et al., 2015; Shoskes et al., 2016; Thomas-White et al., 2016a,b). The main urinary bacteria in healthy women are *Lactobacillus, Prevotella*, and *Gardnerella* (Siddiqui et al., 2011). Although patients with urinary urgency incontinence have been shown to have higher levels of *Gardnerella* and *Lactobacillus gasseri* than controls, they have also been shown to have lower levels of *Lactobacillus crispatus* (Pearce et al., 2014). Women undergoing surgery for stress urinary incontinence have been shown to have increased urinary bacterial diversity, and this diversity has been shown to be affected by hormone status (there were differences between premenopausal women, and postmenopausal women with or without current exogenous hormone use), body mass index, and the symptoms of stress urinary incontinence (Thomas-White et al., 2016b). In addition, the urinary microbiota has been demonstrated to be affected by age. For example, the urinary microbiota of men and women who were 70 years old or over were dominated by *Jonquetella, Parvimonas, Proteiniphilum*, and *Saccharofermentans* (Lewis et al., 2013). Elderly healthy females had significantly lower relative abundance of *Lactobacillus* than non-elderly (Liu et al., 2016).

Studies have demonstrated that individuals with T2DM, and those with comorbid conditions such as hypertension and hyperlipidemia, have alterations in their intestinal microbiota profiles. The risk of developing T2DM and hyperlipidemia has been reported to be correlated with an alteration in the composition and the function of the intestinal microbiota (Suez et al., 2016). For instance, functional analysis demonstrated that the microbiota of T2DM patients had decreased pathways of bacterial chemotaxis, flagellar assembly, butyrate biosynthesis and metabolism of cofactors and vitamins (Qin et al., 2012).

The occurrence of T2DM may also be associated with changes in urinary microbiota profiles, as having diabetes can change the microenvironment of the urinary tract in which bacterium live. For example, those with diabetes can have increased levels of glucose in their urine as a result of their high levels of blood glucose. These high levels of blood glucose can progressively damage the glomeruli, reducing their ability to filtrate blood and causing protein to leak out of the kidneys into the urine.

Hypertension may also be associated with changes in urinary microbiota profiles as it is correlated with an increase in the activity of the renin-angiotensin-aldosterone system, which affects the amount of water excreted by the kidneys (Basi et al., 2008). In addition, higher sodium and lower potassium in the urine are more prevalent in those with hypertension than in those without hypertension (Kieneker et al., 2014; Mente et al., 2016). Furthermore, impaired kidney function is associated with hyperlipidemia (Chen et al., 2016), which can cause high levels of proteinuria (Gordillo and Spitzer, 2009). Thus, the co-occurrence of hypertension and/or hyperlipidemia with T2DM can affect the composition of urine, which can then affect the microenvironment of the urinary tract and consequently alter bacterial growth.

So far, no study has explored the relationship between the urinary microbiota and comorbidities in those with T2DM. We speculate that the composition changes in urine caused by comorbid hypertension, hyperlipidemia and urinary tract infections in female T2DM patients can alter the urinary microbiota profile. Thus, the aim of this study was to investigate the changes in the urinary microbiota profiles of women with T2DM only and those with T2DM plus hypertension and/or hyperlipidemia, and the correlation between the profiles and patient characteristics, i.e., fasting blood glucose (FBG), blood pressure (BP), blood lipids (BL), asymptomatic bacteriuria (according to urine cultures) and nitrite-positive samples (which are indicative of urinary tract infections).

## Materials and methods

### Recruitment of subjects

Seventy individuals with T2DM were divided into four cohorts: 28 were allocated to the T2DM only (DM) cohort, 24 to the T2DM plus hypertension (DM+HT) cohort, 7 to the T2DM plus hyperlipidemia (DM+HLP) cohort, and 11 to the T2DM plus both hypertension and hyperlipidemia (DM+HT+HLP) cohort. The participants had been previously diagnosed (with T2DM only or with T2DM plus hypertension and/or hyperlipidemia) by clinicians in local provincial or municipal hospitals in China. They were enrolled at the Endocrinology Department of the First Affiliated Hospital, School of Medicine, Zhejiang University from June 2015 to January 2016. Written informed consent was obtained from the participants prior to enrollment, and approval for the study was obtained from the Ethics Committee of the First Affiliated Hospital (reference number: 295). The following exclusion criteria were applied: a urinary tract infection (UTI) in the previous month, antibiotic use in the previous 3 months, inability to complete the questionnaire, menstruation, urinary incontinence, known anatomical abnormalities of the urinary tract (e.g., cystoceles, hydronephrosis, renal atrophy, or neurogenic bladder), and use of a urinary catheter. Each participant's most recent FBG, BP, and BL values were obtained from their medical records. Water intake was assessed using the Chinese Food Frequency Questionnaire (Zhao et al., 2010).

### Collection and processing of urinary samples

The samples collected contained the first urine of the day. The participants were taught to use a modified midstream urine (MMSU) collection technique that involved disinfection and a four-tube collection method. The MMSU collection technique involved the following steps: (a) A polyvinylpyrrolidone-iodine antiseptic (Dian'erkang, Shanghai, China) was poured onto sterile cotton balls, which were then placed into a 40 mL sterile sputum cup. (b) Four 50-mL sterile centrifuge tubes (which were labeled tube 1, 2, 3, and 4) were opened and the lids were placed upwards so that the participant would not touch the interior or top edge of either the tubes or the lids. (c) The participant pulled her underwear down to her knees and squatted on a squat toilet, with legs spread apart. (d) The participant disinfected the thumb, middle finger, and index finger of each hand twice with the polyvinylpyrrolidone-iodine antiseptic. (e) The participant used her dominant hand to pick up one of the cotton balls covered with antiseptic, cleaned the far labial fold, starting from above the urinary meatus down toward the rectum. The cotton ball was then discarded without crossing the sterile field that had previously been disinfected. The near labial fold was then disinfected by cleaning down the center of the urinary meatus. The labia were held apart to prevent the labia minora from falling back over the urinary meatus. (f) After disinfection, the participant urinated into tube 1 until half the tube was filled. Then, without stopping the flow of urine, the participant urinated into tubes 2, 3, and 4 in that order. The redundant urine was voided to the toilet. All the tubes were filled up to the halfway mark except for tube 2, which was filled up to the labeled line (representing 40 mL of urine). After training, we asked the participant demonstrating the MMSU technique to assess their competency to perform the procedure. The collection procedure was supervised by a senior nurse.

Each sample was given an anonymous identification code. The urine in tubes 2 and 3 was separated into three aliquots: 15 mL for urinalysis, 1 mL for urine culture, and 40 mL for bacterial DNA sequencing. The urine in tubes 1 and 4 was discarded, which could guarantee that genuine mid-stream urine was used in the experiment. The urinalysis involved testing the sample for nitrites. The urine cultures were performed by inoculating blood agar plates and incubating them at 37°C for 48 h. Asymptomatic bacteriuria was defined as the presence of ≥10^5^ CFU/mL of the same bacterial strain in two consecutive MMSU specimens (Varli et al., 2012). If asymptomatic bacteriuria was diagnosed, urine from the second MMSU specimen was used for bacterial DNA sequencing. A self-report questionnaire was used to collect demographic characteristics.

### DNA extraction, PCR, and MiSeq sequencing

Forty mL urine was aspirated from tubes 2 and 3, separated into three sections and injected into three 15 mL sterile centrifuge tubes. Each of them was pelleted by centrifugation at 4,000 × *g* for 15 min at 4°C. Ten milliliter of the supernatant was decanted and the pellet was obtained by centrifugation for 15 min at 4,000 × *g* at 4°C. The pellet was injected into a 2 mL sterile centrifugation tube which contained 500 μL of lysis buffer, which was composed of 1 M Tris-HCl (pH 8.03), NaCl, 0.5 M EDTA (pH 7.97), and SDS. The tube was kept at −80°C until DNA extraction. The method of magnetic beads isolation of genomic DNA from bacteria was based on the manufacturer's protocol with minor modifications (Shoskes et al., 2016). The tube was placed in liquid nitrogen for 1 min, and transferred into a water bath at 65°C for 5 min, with vigorous mixing. This last process was repeated three times with a final maintenance in the water bath for 30 min. 50 μL Agencourt AMPure XP (Beckman Coulter, USA) was added to 100 μL of the urine pellet, vortexed for 30 s and incubated for 5 min at room temperature. The tube was placed into a magnetic separator for 5 min, and DNA was bound to magnetic beads which were drawn to the wall of the microcentrifuge tube. The supernatant was carefully removed without disrupting the magnetic beads. The sample was washed twice with 200 μL 80% ethanol for 30 s, being placed on a magnet separator between each washing. The purified DNA was eluted with 50 μL ddH_2_O for 1 min. The beads, now released from the DNA, were collected with the magnet, The DNA-containing supernatant was transferred to a clean tube. The concentration of extracted DNA was determined by using a NanoDrop ND-1000 spectrophotometer (Thermo Electron Corporation, USA); its integrity and size were checked by 1.0% agarose gel electrophoresis containing 0.5 mg/mL ethidium bromide. The DNA complex was placed at −20°C until PCR amplification. We failed to extract sufficient DNA for sequencing from the sample of one participant, so this participant was replaced by another who had similar demographic characteristics.

The 16S rRNA gene V3-V4 regions were PCR-amplified from microbial genome DNA using primers (forward primer, 5′-ACTCCTACGGGAGGCAGCAG-3′; reverse primer, 5′-GGACTACHVGGGTWTCTAAT-3′; Fadrosh et al., 2014). All crucial steps during sample processing, DNA isolation and the entire PCR set up were performed in a laminar air flow bench, illuminated with a UV lamp prior to use in order to avoid possible contaminants. In addition, negative DNA extraction controls (lysis buffer and kit reagents only) were amplified and sequenced as contamination controls. The amplicons were normalized, pooled and sequenced on the Illumina MiSeq instrument using a 300 × 2 V3 kit.

### Bioinformatics analysis and statistical analysis

Sequence reads processing was performed using QIIME (version 1.9.0) (Caporaso et al., 2010) and included additional quality trimming, demultiplexing, and taxonomic assignments. Profiling of predictive urinary microbiota was analyzed by using PICRUSt based on the Greengenes database as of 13 August 2013 (DeSantis et al., 2006) (BioProject number: 329477; Accessing number: SRP087709). Specifically, the processed sequences were subjected to subsampled open-reference operational taxonomic unit (OTU) picking against Greengenes, clustering unmatched reads with 97% identity into OTUs. QIIME was used to calculate the Shannon-Weiner indices, and phylogenetic (alpha) diversity. The diversity and richness of the bacteria in the urine samples were calculated using several estimates. These consisted of the level of OTUs, which provides a measure of bacterial richness), Chao1 (which is also an estimate of bacteria richness) and the Shannon and Simpson indices (which are measures of bacterial diversity).

Statistical analysis was performed using the SPSS data analysis program (version 21.0). For continuous variables, Independent *t*-tests and Mann-Whitney *U*-test was applied. For categorical variables between groups, using either the Pearson chi-square or Fisher's exact test, depending on assumption validity. For taxon between cohorts, Mann-Whitney *U*-test was applied. All tests of significance were two sided, and *p* < 0.05 was considered statistically significant. FDR was used as a correction approach to control the false discovery rate (Shoskes et al., 2016). The receiver operating characteristic curve was plotted and the area under curve of each predictive model was calculated using SPSS.

## Results

### Cohort description

Table 1 displays the baseline characteristics of the participants in each cohort. The mean age in the DM+HT cohort was significantly greater than those in the DM and DM+HLP cohorts, the mean age in the DM+HT+HLP cohort was significantly greater than those in the DM and DM+HLP cohorts. The duration of T2DM was significantly shorter in the DM and DM+HLP cohorts than in the DM+HT cohort. Those in the DM+HT cohort were also less likely to be premenstrual compared to those in the DM cohort.

**Table 1:** **Characteristics of the DM, DM+HT, DM+HLP, and DM+HT+HLP cohorts^a^**.

**Characteristic^b^**	**DM (*n* = 25)**	**DM+HT (*n* = 24)**	**DM+HLP (*n* = 7)**	**DM+HT+HLP (*n* = 11)**
Age	56.28 ± 13.91^*^^,^^¥^	70.42 ± 9.00^*^^,^^$^	54.43 ± 10.66^$^^,^^θ^	69.81 ± 9.64^¥^^,^^θ^
Body mass index (kg/m^2^)	23.74 ± 23.41	23.41 ± 4.13	23.63 ± 3.91	25.31 ± 3.48
**MENSTRUAL STATUS (%)**				
Premenopausal	28.00^*^	0.00^*^	14.29	9.09
Postmenopausal	64.00	100.00	71.43	90.91
Hysterectomy	8.00	0.00	14.29	0.00
**MARITAL STATUS (%)**				
Living with partner	96.00	79.17	100.00	90.91
Divorced	4.00	8.33	0.00	0.00
Widowed	0.00	12.50	0.00	9.09
**TOBACCO USE (%)**				
Never	96.00	100.00	100.00	0.00
Occasionally	4.00	0.00	0.00	0.00
Frequently	0.00	0.00	0.00	0.00
**ALCOHOL USE (%)**				
Never	68.00	87.50	0.00	90.91
Occasionally	28.00	8.33	0.00	9.09
Frequently	4.00	4.17	0.00	0.00
Fasting blood glucose (mmol/L)	7.79 ± 2.04	7.63 ± 1.74	8.26 ± 5.24	8.40 ± 1.94
Duration of diabetes (years)	6.92 ± 5.60^*^	14.42 ± 8.75^*^^,^^$^	5.71 ± 4.79^$^	9.09 ± 6.07
Urinary tract infections over the previous year^c^	0.52 ± 0.71	0.63 ± 1.24	0.70 ± 0.49	1.00 ± 1.90
Asymptomatic bacteriuria (%)	1.43	4.29	0.00	2.86
Positive for nitrites (%)^c^	0.00	5.71	0.00	2.86
Systolic/diastolic blood pressure (mmHg)	N/A	142.29 ± 11.59/85.67 ± 7.61	N/A	144.55 ± 8.77/85.82 ± 12.05
Triglycerides (mmol/L)	N/A	N/A	3.37 ± 1.80	2.15 ± 0.43
Low-density lipoprotein cholesterol (mmol/L)	N/A	N/A	3.08 ± 2.38	3.34 ± 0.54
High-density lipoprotein cholesterol (mmol/L)	N/A	N/A	2.50 ± 1.43	2.04 ± 0.60
Total cholesterol (mmol/L)	N/A	N/A	5.05 ± 1.05	5.56 ± 0.81

a *DM, diabetes mellitus; HLP, hyperlipidemia; HT, hypertension; N/A, not applicable*.

b *Independent t-test and Pearson's chi-square test were used to test for significant differences (p < 0.05) in each variable between the four cohorts*.

c *The population with “urinary tract infections over the previous year” and “positive for nitrites” came from general population who have not been diagnosed with urinary tract infection in the last month and had not been previously diagnosed with urinary incontinence*.

* *Represents a significant difference between the DM and DM+HT cohorts*;

¥ *Represents a significant difference between the DM and DM+HT+HLP cohorts*;

$ Represents significant difference between the DM+HT and DM+HLP cohorts; and

θ *Represents a significant difference between the DM+HLP and DM+HT+HLP cohorts*.

### Sequencing data

We obtained 5,175,509 raw sequences from the 70 samples, with a median read length of 2 × 300 and 443 base pairs. After filtering and removing the chimeric sequences, we obtained 3,981,519 reads for further analysis, which accounted for 76.93% of the valid reads, with a mean of 56,878.84 reads (ranging from 13,419 to 146,528) per sample. The mean read length was 438 bp (ranging from 423 to 436). The value of Good's coverage estimator was 98%.

As shown in Table 2, the numbers of reads and OTUs were significantly higher in the DM+HT cohort compared to in the DM and DM+HLP cohorts. In addition, the number of OTUs was dramatically higher in the DM+HT+HLP cohort compared to in the DM+HLP cohort.

**Table 2:** **Richness and diversity estimators in the DM, DM+HT, DM+HLP, and DM+HT+HLP cohorts^a^**.

**Parameter^b^**	**DM**	**DM+HT**	**DM+HLP**	**DM+HT+HLP**
Number of reads	48522.68 ± 23491.16^*^	67119.25 ± 37614.50^*^^,^^¥^	36055.57 ± 23166.08^¥^	64456.27 ± 33866.88
Number of OTUs^a^^,^^b^	40247.36 ± 18946.19^*^	57655.67 ± 32647.91^*^^,^^¥^	28891.14 ± 17367.94^¥^^,^^$^	55127.36 ± 28204.73^$^
ACE^a^	4421.07 ± 2466.79	3382.38 ± 2102.71	3874.37 ± 2606.88	3577.61 ± 2128.65
Chao1	4122.27 ± 2315.01	3099.59 ± 1875.36	3528.80 ± 2304.51	3188.44 ± 1842.75
Shannon index	3.99 ± 2.21	3.95 ± 2.61	5.21 ± 2.22	4.41 ± 1.79
Simpson index	0.65 ± 0.22	0.61 ± 0.28	0.76 ± 0.24	0.75 ± 0.23

a *ACE, abundance-based coverage estimators; DM, diabetes mellitus; HLP, hyperlipidemia; HT, hypertension; OTU, operational taxonomic units*.

b *The operational taxonomic units (OTUs) were defined based on a similarity threshold of 97%; Independent t-test was used to test for significant differences (p < 0.05) in each variable between the four cohorts*.

*, ¥, and $ *indicate significant differences between the DM and DM+HT cohorts, between the DM+HT and DM+HLP cohorts, and between the DM+HLP and DM+HT+HLP cohorts, respectively*.

*The parameters were calculated using QIIME software*.

### Comorbid conditions with T2DM were associated with alterations in the urinary microbiota profiles

The numbers of bacterial phyla detected in the DM, DM+HT, DM+HLP, and DM+HT+HLP cohorts were 40, 37, 30, and 26, respectively. The predominant bacterial phyla in the DM cohort were Proteobacteria (58.27%), Firmicutes (20.83%), Bacteroidetes (10.14%), Actinobacteria (7.52%), and Fusobacteria (1.06%). The DM+HT cohort was dominated by Proteobacteria (60.18%), Firmicutes (20.60%), Bacteroidetes (11.20%), Actinobacteria (5.36%), and Synergistetes (0.54%). The DM+HLP cohort was dominated by Proteobacteria (50.26%), Firmicutes (24.43%), Bacteroidetes (13.36%), Actinobacteria (9.21%), and Acidobacteria (1.18%). The DM+HT+HLP cohort was dominated by Proteobacteria (37.29%), Firmicutes (22.78%), Bacteroidetes (22.47%), Actinobacteria (15.33%), and Fusobacteria (0.82%).

The numbers of bacterial genera in the DM, DM+HT, DM+HLP, and DM+HT+HLP cohorts were 320, 303, 236, and 225, respectively. The genera detected in each of the cohorts are shown in Table S1. Of the genera in the DM cohort, 61, 103, and 108 were not detected in the DM+HT, DM+HLP, and DM+HT+HLP cohorts, respectively. Of the genera in the DM+HT cohort, 41, 86, and 102 were not found in the DM, DM+HLP, and DM+HT+HLP cohorts, respectively. Of the genera in the DM+HLP cohort, 26, 6, and 60 were absent from the DM, DM+HT, and DM+HT+HLP cohorts, respectively. Finally, of the genera in the DM+HT+HLP cohort, 23, 26, and 53 were not present in the DM, DM+HT, and DM+HT+HLP cohorts.

**Figure 1:**
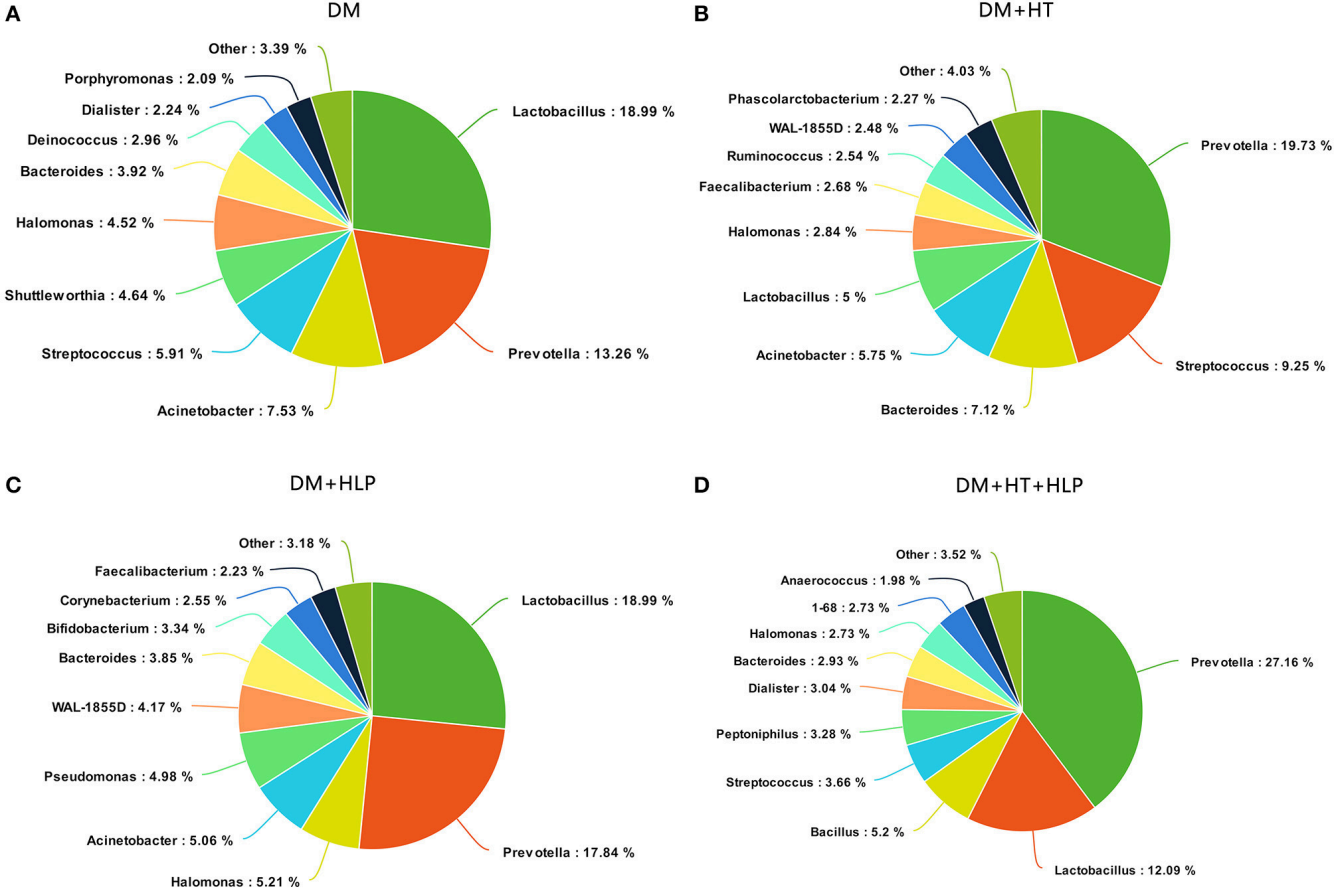
**Summary of bacterial genera detected in the four cohorts. (A–D)** indicate the top 10 most abundant genera detected in the DM, DM+HT, DM+HLP, and DM+HT+HLP cohorts, respectively. DM, diabetes mellitus; HLP, hyperlipidemia; HT, hypertension.

The predominant genera in the DM cohort were *Lactobacillus, Prevotella*, and *Acinetobacter* (Figure 1A). The DM+HT cohort was dominated by *Prevotella, Streptococcus*, and *Bacteroides*, and *Lactobacillus* only accounted for 5% of the DNA sequences (Figure 1B). The samples from the DM+HLP cohort primarily consisted of *Lactobacillus, Prevotella*, and *Halomonas* (Figure 1C). The most common genera in the DM+HT+HLP cohort were *Prevotella, Lactobacillus*, and *Bacillus* (Figure 1D).

**Figure 2:**
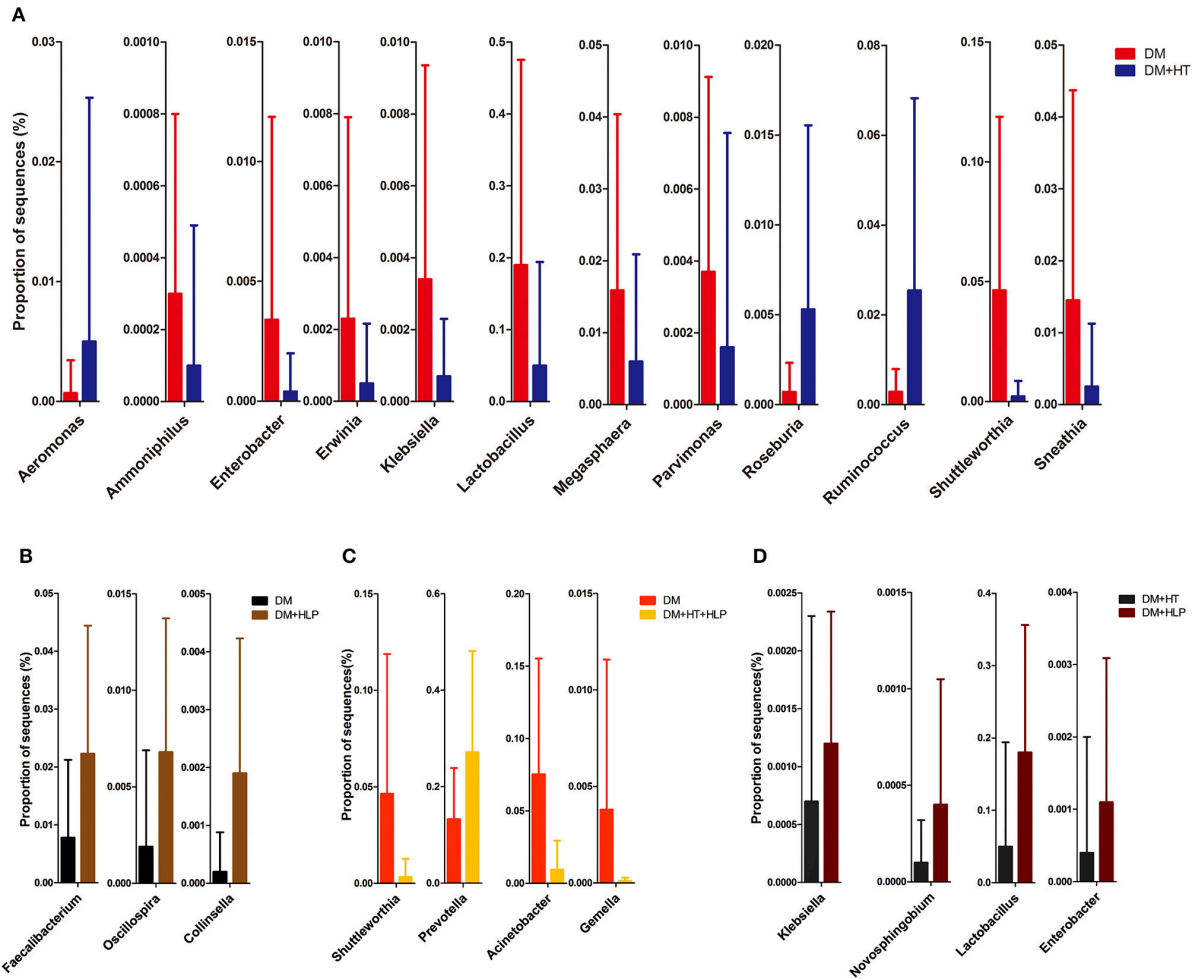
**Genus-level operational taxonomic units that were significantly different between the four cohorts**. Mann-Whitney *U*-tests were used to compare the differences in the relative abundance of bacterial genera between pairs of cohorts. **(A–D)** indicate the significant differences (*p* < 0.05) between the DM and DM+HT cohorts, between the DM and DM+HLP cohorts, between the DM and DM+HT+HLP cohorts, and between the DM+HT and DM+HLP cohorts, respectively. DM, diabetes mellitus; HLP, hyperlipidemia; HT, hypertension.

**Figure 3:**
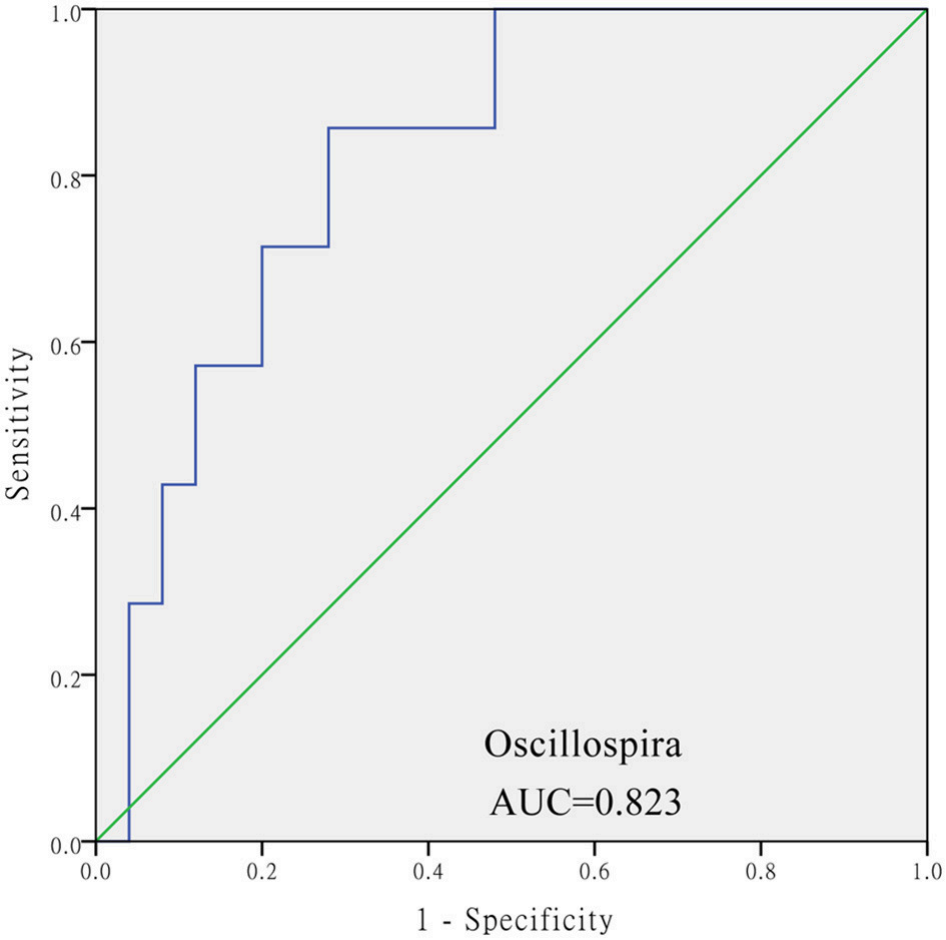
**Receiver operating characteristic curve analysis of sensitivity and specificity of the taxon sequence**. The analysis evaluated the use of the relative abundance of *Oscillospira* as a diagnostic factor for distinguishing between those with diabetes only from those with diabetes plus hyperlipidemia. SPSS software was used for the ROC curve analysis. The diabetes only was labeled with “0” and the diabetes plus hypertenstion was labeled with “1,” and the value of state variable was set “1.”

The levels of *Aeromonas, Roseburia*, and *Ruminococcus* were significantly lower in the DM cohort compared to in the DM+HT cohort, while *Lactobacillus, Enterobacter, Klebsiella*, etc. were dramatically higher in the DM cohort compared to in the DM+HT cohort (Figure 2A). Compared to the DM cohort, the relative abundances of *Faecalibacterium, Oscillospira*, and *Collinsella* were significantly higher in the DM+HLP cohort (Figure 2B). In addition, compared to in the DM cohort, the relative abundance of *Prevotella* was significantly higher in the DM+HT+HLP cohort, while those of *Shuttleworthia, Acinetobacter*, and *Gemella* were dramatically lower in the DM+HT+HLP cohort (Figure 2C). The levels of *Klebsiella, Novosphingobium, Lactobacillus*, and *Enterobacter* were significantly lower in the DM+HT cohort compared to in the DM+HLP cohort (Figure 2D). The relative abundance of *Faecalibacterium* was significantly higher in the DM+HLP cohort compared to in the DM+HT+HLP cohort (0.02 ± 0.02 vs. 0.01 ± 0.01, *p* = 0.040). In addition, as shown in Figure 3, the receiver operating characteristic curve analysis demonstrated that *Oscillospira* was capable of being used as a diagnostic factor for distinguishing those in the DM+HLP cohort from those in the DM cohort.

To better understand the relationships between urinary microbiota and individual characteristics such as FBG, BP, and BL, correlation analyses were carried out. The bacteria that were significantly correlated with the three factors are listed in Tables S2–S5. In the DM cohort, the levels of 4 genera were positively correlated with FBG (Table S2). In the DM+HT cohort, the relative abundance of 8 and 14 genera was positively and negatively correlated with systolic BP, respectively. In addition, 15 genera were negatively correlated with diastolic BP in the DM+HT cohort (Table S3). In the DM+HLP cohort, the levels of 15 and 29 genera were positively correlated with triglycerides and high-density lipoprotein cholesterol (HDL-C), respectively. In the DM+HLP cohort, 8 and 10 genera were negatively correlated with low-density lipoprotein cholesterol (LDL-C) and total cholesterol, respectively (Table S4). In the DM+HT+HLP cohort, the levels of 11 and 1 genera were positively and negatively correlated with systolic and diastolic BP, respectively. Thirteen genera were negatively correlated with triglycerides, 37 were positively correlated with LDL-C, 2 were correlated with HDL-C (one was positively correlated and the other was negatively correlated), and 5 and 10 genera were positively and negatively correlated with total cholesterol (Table S5). No genera were correlated with FBG in any of the cohorts.

At the species level, those in the DM+HT cohort had significantly lower relative abundances of *Lactobacillus iners, Acinetobacter rhizosphaerae*, and *Acinetobacter schindleri* than those in the DM cohort (2.14 ± 10.23 vs. 9.15 ± 22.36, *p* = 0.001; 0.03 ± 0.08 vs. 0.16 ± 0.19, *p* = 0.001; 0.00 ± 0.00 vs. 0.01 ± 0.03, *p* = 0.019, respectively). In contrast, they also had a higher relative abundance of *Kocuria palustris* compared to those in the DM cohort (0.01 ± 0.03 vs. 0.00 ± 0.0, *p* = 0.042). Interestingly, *Eggerthella lenta*, which was not detected in the DM cohort, was found in the DM+HT cohort. In addition, *Deinococcus aquatilis*, which was detected in the DM cohort, was absent in the DM+HT cohort. Additionally, *A. rhizosphaerae* was significantly lower in the DM+HT+HLP cohort compared to in the DM cohort (0.01 ± 0.03 vs. 0.16 ± 0.19, *p* = 0.003). *A. rhizosphaerae* was also significantly lower in the DM+HT+HLP cohort compared to in the DM+HLP cohort (0.00 ± 0.00 vs. 0.06 ± 0.07, *p* = 0.001).

To explore the association between nutrients intake and the relative abundance of bacterial genus in urinary microbiota and between nutrients intake and medicine intake, we compared the difference of nutrients intake among DM, DM+HT, DM+HLP, and DM+HT+HLP group, and there were difference in energy, protein, fat, carbohydrate, vitamin B1, vitamin B2, vitamin C, vitamin E, potassium, sodium, magnesium, selenium, copper, manganese, monounsaturated fatty acid, polyunsaturated fatty acid and aspirin intake among groups. We have found that some nutrients intake were weak or mild positively or negatively associated with urinary microbiota (*r* < 0.7, *p* < 0.05; Tables S6, S7).

## Discussion

Hypertension appears to increase urinary bacterial diversity, as those in the DM+HT and DM+HT+HLP cohorts had larger numbers of reads and/or OTUs compared to those in the DM and DM+HLP cohorts. Hyperlipidemia may lower the diversity of the microbiota, as the DM+HLP cohort had lower numbers of reads and OTUs compared to the DM+HT cohort. The results were similar at both the phylum and genus levels. Among the four cohorts, subjects with hyperlipidemia had a lower presence of bacteria than those without hyperlipidemia.

The top four most abundant bacteria in the four cohorts were (in various orders) Proteobacteria, Firmicutes, Bacteroidetes, and Actinobacteria. This is somewhat similar to the findings of several previous studies on urinary microbiota profiles, as in these previous studies, one of the most abundant bacteria was Proteobacteria, which were generally ranked as the second or third most abundant bacteria, though they sometimes ranked lower (Siddiqui et al., 2011, 2012; Pearce et al., 2014; Karstens et al., 2016; Thomas-White et al., 2016a).

Among the four cohorts, the rates of participants with asymptomatic bacteriuria and nitrite-positive samples were the highest in the DM+HT cohort. Interestingly, among the four cohorts, the relative abundance of Proteobacteria was also the highest in the DM+HT cohort. Lewis et al. (2013) reported that Proteobacteria was the predominant phylum in nitrite-positive samples in their study. The findings in our study and the study by Lewis et al. indicate that the relative abundance of Proteobacteria may be correlated with UTIs. Evidence has shown that hypertension is a risk factor for UTIs in those with diabetes (Al-Rubeaan et al., 2013). This suggests that Proteobacteria may play a role in the high prevalence of UTIs in those with diabetes and comorbid hypertension.

Interestingly, asymptomatic bacteriuria and nitrite-positive samples were not found in the DM+HLP cohort and, among the four cohorts, the DM+HLP cohort had the lowest relative abundance of Proteobacteria. In addition, *Escherichia* which is one of the most frequent uropathogens (Reygaert, 2014), a genus belonging to Proteobacteria, was not detected in the DM+HLP cohort (Table S1). This suggests that the absence of asymptomatic bacteriuria and nitrite-positive samples in the DM+HLP cohort may be due to the low relative abundance of Proteobacteria and the absence of *Escherichia* in the urine. This may indicate that there is a microbiota-related regulating system that prevented those in the DM+HLP cohort from developing UTIs. This is consistent with the finding that, although the co-occurrence of hyperlipidemia and diabetes can increase the body's inflammatory response (Casqueiro et al., 2012; Chen S. et al., 2014), there were no indications of UTIs (by either asymptomatic bacteriuria or nitrite-positive samples) in the DM+HLP cohort.

Several bacteria were not present in the DM+HLP cohort, but were detected in the three other cohorts such as *Gardnerella*, which has been reported to be a major urotype in previous studies (Thomas-White et al., 2016a,b). Price et al. found that *Gardnerella* was present more often in non-UTI samples than in UTI samples (Price et al., 2016), but in the present study, there were no participants with either asymptomatic bacteriuria or nitrite-positive samples in the DM+HLP cohort. Notably, *Akkermansia* and *Bifidobacterium*, which are considered probiotic bacteria, were detected in all four cohorts. In a previous intestinal microbiota study, the relative abundance of *Akkermansia* was lower in patients with inflammatory diseases compared to healthy humans, and was also lower in diabetic mice (Dingemanse et al., 2015). *Bifidobacterium* has been shown to be able to reduce levels of intestinal endotoxins and improve the mucosal barrier, which results in reduced systemic inflammation (Cani et al., 2008). Although T2DM, hypertension, and hyperlipidemia can increase an individual's inflammatory response (Casqueiro et al., 2012; Mangin, 2014; Chen S. et al., 2014), the subjects in the DM, DM+HT, and DM+HLP cohorts did not have a higher incidence of UTIs (in the previous year) compared to the incidence of UTIs (0.5–0.7 per year) reported by Hooten et al. in healthy women (Hooton et al., 1996). This may be due to the presence of bacteria in urine (such as *Akkermansia* and *Bifidobacterium*) that help to combat the inflammatory responses caused by diabetes, hypertension, and hyperlipidemia in order to maintain healthy urinary conditions.

The most abundant bacteria in the DM and DM+HLP cohorts was *Lactobacillus*, which is similar to the findings of previous studies (Siddiqui et al., 2011, 2012; Pearce et al., 2014, 2015; Thomas-White et al., 2016b). However, *Lactobacillus* was the fifth most abundant bacteria and only accounted for 5% of the DNA sequences in the DM+HT cohort, and the level of *Lactobacillus* was significantly lower in the DM+HT cohort compared to in the DM cohort. The relative abundance of *Lactobacillus* may have been affected by the participants' ages, as the participants in the DM+HT and DM+HT+HLP cohorts were significantly older than those in the DM and DM+HLP cohorts. Thomas-White et al. reported a similar finding in their study of urgency incontinence patients, i.e., the urgency incontinence patients were significantly older than the healthy controls, and they had a significantly lower proportion of *Lactobacillus* in their urine (Thomas-White et al., 2016a). Moreover, the DM+HT cohort included fewer premenopausal women than the DM cohort. A recent study demonstrated that premenopausal women and postmenopausal women taking exogenous hormones had higher levels of *Lactobacillus* in their urine compared to postmenopausal women who were not taking exogenous hormones (Thomas-White et al., 2016b). In addition, another study reported that *Lactobacillus* was a dominant genus in urine samples from premenopausal women (Karstens et al., 2016). Although age and menopausal status play a role in the relative abundance of *Lactobacillus*, hypertension may also be a risk factor for reduced levels of *Lactobacillus*. The DM+HT cohort did not include a significantly greater number of postmenopausal women than the DM+HLP cohort, but the relative abundance of *Lactobacillus* was still significantly lower in the DM+HT compared to in the DM+HLP cohort. Furthermore, it has been reported that oral administration of recombinant *Lactobacillus* can significantly decrease systolic BP (Yang et al., 2015). Future studies should explore whether a deficiency in *Lactobacillus* is responsible for the co-occurrence of hypertension in those with diabetes or whether the co-occurrence of hypertension causes the low relative abundance of *Lactobacillus* in urine. Notably, among the four cohorts, the DM+HT subjects had the highest rates of asymptomatic bacteriuria and nitrite-positive samples and lowest relative abundance of *Lactobacillus*. Hypertension is a risk factor for UTIs in diabetic patients (Al-Rubeaan et al., 2013) and *Lactobacillus* can reduce UTIs (Marelli et al., 2004).

Interestingly, not only was the relative abundance of *Oscillospira* dramatically higher in the DM+HLP cohort compared to in the DM cohort, but it was also capable of being used as a diagnostic factor for differentiating those in the DM+HLP cohort from those in the DM. If this finding is confirmed by a study with a larger sample size, the relative abundance of *Oscillospira* could be used as a measure for patients with diabetes to self-monitor hyperlipidemia.

The relative abundance of *Faecalibacterium* was higher in the DM+HLP cohort than in the DM cohort. It has been reported that patients with urgent urinary incontinence also have a higher proportion of *Faecalibacterium* than healthy controls (Pearce et al., 2014), and hyperlipidemia has been shown to increase urinary frequency and decrease the density of blood vessels and nerves in the bladder (Huang et al., 2010). This suggests that reducing the relative abundance of *Faecalibacterium* in urine may be useful for controlling hyperlipidemia in patients with urgent urinary incontinence.

There was a significantly higher relative abundance of *Prevotella* in the DM+HT+HLP cohort compared to in the DM cohort. In addition, among the four cohorts, the DM cohort had the lowest relative abundance of *Prevotella*. A previous study reported that the relative abundance of *Prevotella* was higher in patients with urgency urinary incontinence compared to healthy controls (Pearce et al., 2014), and compared to healthy controls, those with urgency urinary incontinence had a significantly higher mean body mass index (which is a good predictor of hyperlipidemia) (Rao et al., 2016), and a greater degree of hypertension (Pearce et al., 2014). Therefore, the high relative abundance of *Prevotella* may be a causal factor in the development of hypertension and hyperlipidemia.

At the genus level, we found that several bacteria correlated with FBG, BP, and BL. *Atopobium* was positively correlated with FBG in the DM cohort, which conflicts with the findings of a recent intestinal study in which the relative abundance of *Atopobium* was lower in those with diabetes compared to in healthy controls (Sato et al., 2014). *Allobaculum* was negatively correlated with diastolic BP in the DM+HT cohort. An intestinal microbiota study revealed that mice eating a high fat diet (which is associated with high BP) had a lower relative abundance of *Allobaculum* than controls (Ravussin et al., 2011). Therefore, reducing the relative abundance of *Allobaculum* in urine may be useful for controlling BP if the association between urinary *Allobaculum* and BP is similar to that between intestinal *Allobaculum* and BP. The relative abundance of *Rikenella* was positively correlated with triglycerides in the DM+HLP cohort. This finding is similar to the findings of a previous study that detected *Rikenella* in the ceca of diabetic mice with dyslipidemia (Harris et al., 2012). In addition, a recent study of intestinal microbiota revealed that the relative abundance of *Odoribacter* is negatively correlated with systolic BP (Gomez-Arango et al., 2016), which is somewhat similar to the findings in the present study, as the relative abundance of *Odoribacter* in the DM+HT+HLP cohort was negatively associated with diastolic BP. Moreover, Chen et al. reported a positive correlation between *Dorea* and LDL-C in a rat model of hyperlipidemia (Chen D. et al., 2014), and *Dorea* was also positively associated with LDL-C in the DM+HT+HLP cohort in the present study.

The relative abundance of *L. iners*, which has been reported to be the most abundant sequence in the urine of patients with sexually transmitted infections (Nelson et al., 2010), was dramatically higher in the DM+HT cohort than in the DM cohort. Several studies have detected *L. iners* in people with infections, indicating that it does not protect against infections (Saunders et al., 2007; Tamrakar et al., 2007). This corresponds with the fact that higher levels of *L. iners* might be correlated with asymptomatic bacteriuria and nitrite-positive samples in the DM+HT cohort. In addition, *E. lenta*, which has been shown to be correlated with the occurrence of bacteremia (Gardiner et al., 2015), was not detected in the DM cohort but was detected in the DM+HT cohort. This suggests that the elevated inflammatory response in those with diabetes plus hypertension may be related to the presence of *E. lenta*. The relative abundance of *A. rhizosphaerae* was lower in the DM+HT+HLP cohort compared to in the DM cohort, and the relative abundance of this bacteria was also lower in the DM+HT+HLP cohort compared to in the DM+HLP cohort, which suggests that hypertension plays a role in inhibiting this species.

The main limitation of the study was that the sizes of the four cohorts were not equal, and some were particularly small, which may bias the results as it can make it more difficult to detect differences between the cohorts. Furthermore, in the DM+HT, DM+HLP, and DM+HT+HLP cohorts, the participants could not recall sequence of the occurrences of their conditions, so we could not use information on the onset of these conditions to determine the influences on the urinary microbiota profiles. In addition, although nutrient intakes were not strongly associated with urinary microbiota and no previous study reported that urinay microbiota were affected by food and supplements intake (Nelson et al., 2010; Dong et al., 2011; Siddiqui et al., 2011, 2012, 2014; Fouts et al., 2012; Wolfe et al., 2012; Fricke et al., 2014; Nienhouse et al., 2014; Pearce et al., 2014, 2015; Price et al., 2016; Shoskes et al., 2016; Thomas-White et al., 2016b), it might skew the results. We would enroll participants who has the same amounts of food and supplements intake in our future study to rule out the effects of food, supplements and medicine intake on urinary microbiota.

## Conclusions

This is the first study to investigate the association between urinary microbiota profiles and comorbid hypertension and hyperlipidemia in those with T2DM. It demonstrated that the co-occurrence of hypertension and hyperlipidemia in those with T2DM affects the relative abundance of various bacteria in urine. UTIs may be affected by the relative abundances of Proteobacteria, *Lactobacillus*, and *L. iners*. FBG, BP, and BL were correlated with the relative abundances of several bacteria. The urinary system is a vital excretory system for metabolic waste and it plays a crucial role in regulating metabolic diseases. The urinary microenvironment (which is produced by the composition of urine) has a large impact on the reproduction of the microflora. Therefore, it is important to further investigate the mechanisms behind the association of urinary microbiota with hypertension, hyperlipidemia, and urinary tract infections, as this may be useful for developing microbiota-based treatment to control hypertension, hyperlipidemia, and bladder infections in those with diabetes.

## Author contributions

LL, WW, YX, and FL conceived and designed the study. ZL generated the sequencing data. FL, LZ, and PJ collected the samples. QY and FL conducted urine cultures and the urinalysis. FL, BW, and LZ extracted the bacterial DNA. FL, ZL, and YX analyzed the data, carried out the computational analysis, interpreted the data, and drafted the manuscript.

## Funding

This study was funded by the Opening Foundation of China's State Key Laboratory for Diagnosis and Treatment of Infectious Diseases (grant number: 2015KF05).

### Conflict of interest statement

The authors declare that the research was conducted in the absence of any commercial or financial relationships that could be construed as a potential conflict of interest.
