# Supplementary material for: Alterations of Urinary Microbiota in Type 2 Diabetes Mellitus with Hypertension and/or Hyperlipidemia
Source: Front Physiol. 2017 Mar 3;8:126. doi: 10.3389/fphys.2017.00126 (PMC5334339; doi:10.3389/fphys.2017.00126)
Supplement: Supplementary file 1 [file Table1.DOC]

**TALBE S1 Bacterial genera in the urine samples from the DM, DM+HT, DM+HLP, and DM+HT+HLP cohorts *^a,b^.***

| **DM** | **DM+HT** | **DM+HLP** | **DM+HT+HLP** |
| --- | --- | --- | --- |
| Abiotrophia *^*1*1,*2,*3^* | Achromobacter | Achromobacter | Achromobacter |
| Achromobacter | Acidaminococcus | Acidaminococcus | Acidaminococcus |
| Acidaminococcus | Acidovorax | Acidovorax | Acidovorax |
| Acidovorax | Acinetobacter | Acinetobacter | Acinetobacter |
| Acinetobacter | Actinobacillus *^¥1,¥3^* | Actinobacillus *^＄1,＄3^* | Actinobaculum |
| Actinobaculum | Actinobaculum | Actinobaculum | Actinomyces |
| Actinomyces | Actinomyces | Actinomyces | Adlercreutzia |
| Adhaeribacter *^*2,*3^* | Adhaeribacter *^¥2,¥3^* | Actinotalea *^＄2,＄3^* | Aerococcus |
| Adlercreutzia | Adlercreutzia | Adlercreutzia | Aeromicrobium |
| Aerococcus | Aerococcus | Aerococcus | Aeromonas |
| Aeromicrobium | Aeromicrobium | Aeromicrobium | Agrobacterium |
| Aeromonas | Aeromonas | Aeromonas | Agromyces |
| Afifella *^*1*1,*2,*3^* | Aggregatibacter *^¥3^* | Aggregatibacter *^＄3^* | Akkermansia |
| Aggregatibacter *^*3^* | Agrobacterium | Agrobacterium | Alcanivorax *^θ2,θ3^* |
| Agrobacterium | Agromyces | Agromyces | Alicyclobacillus *^θ2,θ3^* |
| Agromyces | Akkermansia | Akkermansia | Allobaculum |
| Akkermansia | Alkanindiges *^¥2,¥3^* | Allobaculum | Alloiococcus |
| Alcanivorax *^*1,*2^* | Allobaculum | Alloiococcus | Ammoniphilus |
| Alicyclobacillus *^*1,*2^* | Alloiococcus | Amaricoccus *^＄3^* | Anaerococcus |
| Alkanindiges *^*2,*3^* | Amaricoccus *^¥3^* | Ammoniphilus | Anaerostipes |
| Allobaculum | Aminobacter *^¥2,¥3^* | Anaerococcus | Anoxybacillus |
| Alloiococcus | Ammoniphilus | Anaerostipes | Aquaspirillum |
| Amaricoccus *^*3^* | Amycolatopsis *^¥1,¥2,¥3^* | Anaerotruncus *^＄1,＄3^* | Aquicella *^θ2,θ3^* |
| Aminobacter *^*2,*3^* | Anaerococcus | Anoxybacillus | Arcanobacterium |
| Ammoniphilus | Anaeromyxobacter *^¥2,¥3^* | Aquaspirillum | Arenibacter *^θ1,θ2,θ3^* |
| Anaerobacillus *^*1,*2,*3^* | Anaerostipes | Aquimonas *^＄1,＄3^* | Arthrobacter |
| Anaerococcus | Anaerotruncus *^¥1,¥3^* | Arcanobacterium | Asticcacaulis *^θ1,θ3^* |
| Anaeromyxobacter *^*2,*3^* | Anoxybacillus | Arcobacter *^＄3^* | Atopobium |
| Anaerostipes | Aquaspirillum | Arenimonas *^＄3^* | Azoarcus |
| Aneurinibacillus *^*1,*2,*3^* | Arcanobacterium | Arthrobacter | Azohydromonas |
| Anoxybacillus | Arenimonas *^¥3^* | Atopobium | Azospirillum *^θ1,θ3^* |
| Aquaspirillum | Arthrobacter | Azoarcus | Bacillus |
| Aquicella *^*1,*2^* | Asticcacaulis *^¥1,¥2,¥3^* | Azohydromonas | Bacteroides |
| Arcanobacterium | Atopobium | Bacillus | Balneimonas *^θ3^* |
| Arcobacter *^*1,*3^* | Azoarcus | Bacteroides | Bdellovibrio |
| Ardenscatena *^*1,*2,*3^* | Azohydromonas | Bdellovibrio | Beijerinckia |
| Arenimonas *^*3^* | Azospirillum *^¥1,¥2,¥3^* | Beijerinckia | Bifidobacterium |
| Arsenicicoccus *^*1,*2,*3^* | Bacillus | Bifidobacterium | Bilophila |
| Arthrobacter | Bacteroides | Bilophila | Blastomonas *^θ3^* |
| Atopobium | Balneimonas *^¥2^* | Blautia | Blautia |
| Azoarcus *^*1^* | Bdellovibrio | Bosea | Bosea |
| Azohydromonas | Beijerinckia | Bradyrhizobium | Brachybacterium *^θ3^* |
| Azospira *^*2,*3^* | Bifidobacterium | Brevibacterium | Bradyrhizobium |
| Bacillus | Bilophila | Brevundimonas | Brevibacillus *^θ3^* |
| Bacteroides | Blastomonas *^¥2^* | Bulleidia | Brevibacterium |
| Balneimonas *^*2^* | Blautia | Burkholderia | Brevundimonas |
| Bdellovibrio | Bosea | Butyricimonas | Brochothrix *^θ3^* |
| Beijerinckia | Brachybacterium *^¥2^* | Campylobacter | Bulleidia |
| Bifidobacterium | Bradyrhizobium | Candidatus | Burkholderia |
| Bilophila | Brevibacillus *^¥2^* | Carnobacterium | Butyricimonas |
| Blastomonas *^*2^* | Brevibacterium | Caulobacter *^＄3^* | Campylobacter |
| Blautia | Brevundimonas | Cellulomonas *^＄3^* | Candidatus |
| Bosea | Brochothrix *^¥2^* | Cellvibrio | Carnobacterium |
| Brachybacterium *^*2^* | Bulleidia | Chloronema *^＄1,＄3^* | Cellvibrio |
| Bradyrhizobium | Burkholderia | Christensenella *^＄1,＄3^* | Chryseobacterium |
| Brevibacillus *^*2^* | Butyricimonas | Chryseobacterium | Citrobacter |
| Brevibacterium | Campylobacter | Citrobacter | Cloacibacterium |
| Brevundimonas | Candidatus | Cloacibacterium | Clostridium |
| Brochothrix *^*2^* | Capnocytophaga *^¥2,¥3^* | Clostridium | Collinsella |
| Bulleidia | Carnobacterium | Collinsella | Comamonas |
| Burkholderia | Catenibacterium *^¥1,¥2,¥3^* | Comamonas | Coprobacillus *^θ1,θ2^* |
| Butyricimonas | Caulobacter *^¥3^* | Coprobacillus *^＄1^* | Coprococcus |
| Caldilinea *^*1,*2,*3^* | Cellvibrio | Coprococcus | Corynebacterium |
| Campylobacter | Cetobacterium *^¥1,¥2,¥3^* | Corynebacterium | Cryocola *^θ2^* |
| Candidatus | Christensenella *^¥1,¥3^* | Cryocola | Cupriavidus |
| Capnocytophaga *^*2,*3^* | Chryseobacterium | Cupriavidus | Curtobacterium |
| Carnobacterium | Citrobacter | Curtobacterium | Dehalobacterium |
| Catellatospora *^*1,*2,*3^* | Cloacibacterium | Dechloromonas *^＄3^* | Deinococcus |
| Caulobacter *^*3^* | Clostridium | Dehalobacterium | Delftia |
| Cellulomonas *^*1,*3^* | Collinsella | Deinococcus | Dermabacter *^θ3^* |
| Cellvibrio | Comamonas | Delftia | Dermacoccus |
| Chitinophaga *^*1,*2,*3^* | Coprococcus | Dermacoccus | Desulfovibrio |
| Chryseobacterium | Corynebacterium | Desulfovibrio | Dialister |
| Chthoniobacter *^*1,*2,*3^* | Cupriavidus | Devosia *^＄3^* | Dorea |
| Citrobacter | Curtobacterium | Dialister | Dysgonomonas *^θ1,θ2,θ3^* |
| Cloacibacterium | Cytophaga *^¥1,¥2,¥3^* | Diaphorobacter *^＄3^* | Eggerthella *^θ1,θ3^* |
| Clostridium | Dechloromonas *^¥3^* | Dorea | Enhydrobacter |
| Collinsella | Dehalobacterium | Eggerthella *^＄1^* | Enterobacter |
| Comamonas | Deinococcus | Enhydrobacter | Enterococcus |
| Coprococcus | Delftia | Enterobacter | Erwinia |
| Corynebacterium | Dermabacter *^¥2^* | Enterococcus | Escherichia |
| Cryocola *^*1^* | Dermacoccus | Epulopiscium *^＄3^* | Exiguobacterium |
| Cupriavidus | Desulfobacca *^¥2,¥3^* | Erwinia | Facklamia |
| Curtobacterium | Desulfococcus *^¥2,¥3^* | Eubacterium *^＄3^* | Faecalibacterium |
| Dechloromonas *^*3^* | Desulfovibrio | Exiguobacterium | Fervidobacterium *^θ2^* |
| Dehalobacterium | Devosia *^¥3^* | Facklamia | Finegoldia |
| Dehalogenimonas *^*1,*2,*3^* | Dialister | Faecalibacterium | Flavihumibacter *^θ3^* |
| Deinococcus | Diaphorobacter *^¥3^* | Fervidobacterium | Flavobacterium |
| Delftia | Dietzia *^¥2,¥3^* | Finegoldia | Flectobacillus *^θ3^* |
| Dermabacter *^*2^* | Dorea | Flavisolibacter *^＄3^* | Fluviicola |
| Dermacoccus | Eggerthella *^¥1,¥3^* | Flavobacterium | Friedmanniella *^θ1,θ2,θ3^* |
| Desulfobacca *^*2,*3^* | Enhydrobacter | Fluviicola | Fusobacterium |
| Desulfobulbus *^*1,*2,*3^* | Enterobacter | Fusibacter *^＄1,＄3^* | Gallicola *^θ3^* |
| Desulfococcus *^*2,*3^* | Enterococcus | Fusobacterium | Gardnerella *^θ3^* |
| Desulfovibrio | Epulopiscium *^¥3^* | Gemella | Gemella |
| Devosia *^*3^* | Erwinia | Geobacillus | Gemmata *^θ1,θ2,θ3^* |
| Dialister | Escherichia *^¥2^* | Geodermatophilus *^＄1,＄3^* | Geobacillus *^θ2^* |
| Diaphorobacter *^*3^* | Eubacterium *^¥3^* | Georgenia *^＄1,＄3^* | Granulicatella |
| Dietzia *^*2,*3^* | Exiguobacterium | Giesbergeria *^＄3^* | Haemophilus |
| Dokdonella *^*1,*2,*3^* | Facklamia | Gordonia *^＄1,＄3^* | Haliangium *^θ1,θ2,θ3^* |
| Dorea | Faecalibacterium | Granulicatella | Halomonas |
| Emticicia *^*1,*2,*3^* | Filifactor *^¥2,¥3^* | Haemophilus | Helcococcus |
| Enhydrobacter | Finegoldia | Halomonas | Helicobacter *^θ1,θ3^* |
| Ensifer *^*1,*2,*3^* | Flavihumibacter *^¥2^* | Helcococcus | Hydrogenophaga |
| Enterobacter | Flavisolibacter *^¥3^* | Holdemania *^＄1,＄3^* | Hydrogenophilus |
| Enterococcus | Flavobacterium | Hydrogenophaga | Hylemonella *^θ3^* |
| Epulopiscium *^*3^* | Flectobacillus *^¥2^* | Hydrogenophilus | Hymenobacter *^θ3^* |
| Erwinia | Fluviicola | Hyphomicrobium *^＄3^* | Hyphomonas |
| Escherichia *^*2^* | Fusobacterium | Hyphomonas | Inquilinus *^θ2,θ3^* |
| Eubacterium *^*3^* | Gallicola *^¥2^* | Janthinobacterium | Janthinobacterium |
| Exiguobacterium | Gallionella *^¥1,¥2,¥3^* | Jonquetella | Jeotgalicoccus *^θ1,θ3^* |
| Facklamia | Gardnerella *^¥2^* | Kaistobacter | Jonquetella |
| Faecalibacterium | Gemella | Klebsiella | Kaistobacter |
| Fervidobacterium *^*1^* | Gemmatimonas *^¥2,¥3^* | Kribbella *^＄1,＄2,＄3^* | Klebsiella |
| Filifactor *^*2,*3^* | Geobacter *^¥2,¥3^* | Lachnobacterium | Kocuria *^θ3^* |
| Finegoldia | Geodermatophilus *^¥1,¥3^* | Lachnospira | Lachnobacterium |
| Flavihumibacter *^*2^* | Gluconobacter *^¥1,¥2,¥3^* | Lactobacillus | Lachnospira |
| Flavisolibacter *^*3^* | Gordonia *^¥1,¥3^* | Lactococcus | Lactobacillus |
| Flavobacterium | Granulicatella | Legionella *^＄1^* | Lactococcus |
| Flectobacillus *^*2^* | Haemophilus | Leptotrichia *^＄3^* | Legionella *^θ1,θ2^* |
| Fluviicola | Halomonas | Leucobacter | Leucobacter |
| Fusobacterium | Helcococcus | Listeria | Listeria |
| Gallicola *^*2^* | Helicobacter *^¥1,¥2,¥3^* | Luteimonas | Luteibacter *^θ1^* |
| Gardnerella *^*2^* | Hydrogenophaga | Lysinibacillus *^＄3^* | Luteimonas |
| Gemella | Hydrogenophilus | Lysobacter | Lysobacter *^θ3^* |
| Gemmatimonas *^*2,*3^* | Hylemonella *^¥2^* | Megamonas | Marinibacillus *^θ1,θ2,θ3^* |
| Geobacillus *^*1^* | Hymenobacter *^¥2^* | Megasphaera | Megamonas |
| Geobacter *^*2,*3^* | Hyphomicrobium *^¥3^* | Methylibium | Megasphaera |
| Giesbergeria *^*1,*3^* | Hyphomonas | Methylobacterium | Methylibium |
| Granulicatella | Iamia *^¥2,¥3^* | Methylotenera *^＄3^* | Methylobacterium |
| Haemophilus | Janthinobacterium | Methyloversatilis | Methyloversatilis |
| Haliscomenobacter *^*1,*2,*3^* | Jeotgalicoccus *^¥1,¥2,¥3^* | Microbacterium | Microbacterium |
| Halomonas | Jonquetella | Micrococcus | Micrococcus |
| Helcococcus | Kaistia *^¥2,¥3^* | Microcystis *^＄2,＄3^* | Mobiluncus |
| Hydrogenophaga | Kaistobacter | Mobiluncus | Mogibacterium *^θ1,θ3^* |
| Hydrogenophilus | Klebsiella | Moryella | Morganella *^θ3^* |
| Hylemonella *^*2^* | Knoellia *^¥2,¥3^* | Mycobacterium | Moryella |
| Hymenobacter *^*2^* | Kocuria *^¥2^* | Mycoplana | Mycobacterium |
| Hyphomicrobium *^*3^* | Lachnobacterium | Nevskia *^＄1,＄3^* | Mycoplana |
| Hyphomonas | Lachnospira | Nitrospira | Mycoplasma *^θ3^* |
| Iamia *^*2,*3^* | Lactobacillus | Novosphingobium | Neisseria *^θ3^* |
| Inquilinus *^*1,*2^* | Lactococcus | Ochrobactrum | Nesterenkonia *^θ3^* |
| Janthinobacterium | Lautropia *^¥2,¥3^* | Odoribacter | Nitrospira |
| Jonquetella | Leptolyngbya *^¥2,¥3^* | Oscillospira | Novosphingobium |
| Kaistia *^*2,*3^* | Leptotrichia *^¥3^* | Paenibacillus *^＄3^* | Ochrobactrum |
| Kaistobacter | Leucobacter | Paludibacter *^＄3^* | Odoribacter |
| Klebsiella | Limnohabitans *^¥2,¥3^* | Pantoea *^＄1,＄2,＄3^* | Oscillospira |
| Knoellia *^*2,*3^* | Listeria | Parabacteroides | Paenisporosarcina *^θ2,θ3^* |
| Kocuria *^*2^* | Luteibacter *^¥1,¥2,¥3^* | Paracoccus | Parabacteroides |
| Lachnobacterium | Luteimonas | Paraprevotella | Paracoccus |
| Lachnospira | Lysinibacillus *^¥3^* | Parvimonas | Paraprevotella |
| Lacibacter *^*1,*2,*3^* | Lysobacter | Pedobacter | Parvimonas |
| Lactobacillus | Marinilactibacillus *^¥1,¥2,¥3^* | Pedomicrobium *^＄2^* | Pedobacter |
| Lactococcus | Megamonas | Peptococcus | Pedomicrobium *^θ2^* |
| Lampropedia *^*1,*2,*3^* | Megasphaera | Peptoniphilus | Peptococcus |
| Lautropia *^*2,*3^* | Meiothermus *^¥2,¥3^* | Peptostreptococcus | Peptoniphilus |
| Leptolyngbya *^*2,*3^* | Mesorhizobium *^¥2,¥3^* | Phascolarctobacterium | Peptostreptococcus |
| Leptotrichia *^*3^* | Methylibium | Phenylobacterium *^＄3^* | Phascolarctobacterium |
| Leucobacter *^*1^* | Methylobacterium | Phormidium *^＄3^* | Phyllobacterium |
| Leuconostoc *^*2,*3^* | Methylotenera *^¥3^* | Phyllobacterium | Planomicrobium |
| Limnohabitans *^*2,*3^* | Methyloversatilis | Pilimelia *^＄1,＄3^* | Plesiocystis |
| Listeria | Microbacterium | Pimelobacter *^＄3^* | Porphyromonas |
| Luteimonas | Micrococcus | Planktothrix *^＄3^* | Prevotella |
| Luteolibacter *^*1,*2,*3^* | Mitsuokella *^¥2,¥3^* | Planomicrobium | Promicromonospora *^θ2^* |
| Lysinibacillus *^*3^* | Mobiluncus | Plesiocystis | Propionibacterium |
| Lysobacter | Modestobacter *^¥1,¥2,¥3^* | Polaromonas *^＄3^* | Prosthecobacter *^θ3^* |
| Megamonas | Mogibacterium *^¥1,¥2,¥3^* | Polynucleobacter *^＄3^* | Proteus |
| Megasphaera | Moraxella *^¥1,¥2,¥3^* | Pontibacter *^＄3^* | Providencia *^θ1,θ2,θ3^* |
| Meiothermus *^*2,*3^* | Morganella *^¥2^* | Porphyromonas | Pseudomonas |
| Mesorhizobium *^*2,*3^* | Moryella | Prevotella | Pseudoxanthomonas |
| Methylibium | Mycobacterium | Promicromonospora | Pyramidobacter |
| Methylobacterium | Mycoplana | Propionibacterium | Ralstonia *^θ3^* |
| Methylocaldum *^*1,*2,*3^* | Mycoplasma *^¥2^* | Proteus | Ramlibacter |
| Methylotenera *^*3^* | Neisseria *^¥2^* | Pseudomonas | Rhodococcus |
| Methyloversatilis | Nesterenkonia *^¥2^* | Pseudonocardia *^＄3^* | Rhodoplanes |
| Microbacterium | Nitrospira | Pseudoramibacter_Eubacterium *^＄1,＄3^* | Rikenella *^θ1^* |
| Micrococcus | Nocardioides *^¥1,¥2,¥3^* | Pseudoxanthomonas | Roseateles *^θ2,θ3^* |
| Microcystis *^*1,*3^* | Novosphingobium | Pyramidobacter | Roseburia |
| Mitsuokella *^*2,*3^* | Ochrobactrum | Ramlibacter | Roseomonas *^θ3^* |
| Mobiluncus | Odoribacter | Rhodobacter *^＄3^* | Rothia |
| Morganella *^*2^* | Oribacterium *^¥1,¥2,¥3^* | Rhodococcus | Rubellimicrobium *^θ3^* |
| Moryella | Oscillospira | Rhodoferax *^＄3^* | Ruminococcus |
| Mycobacterium | Oxalobacter *^¥2,¥3^* | Rhodoplanes | Sediminibacterium |
| Mycoplana | Paenibacillus *^¥3^* | Rickettsia *^＄1,＄3^* | Selenomonas |
| Mycoplasma *^*2^* | Paludibacter *^¥3^* | Rikenella *^＄1^* | Serratia |
| Myroides *^*1,*2,*3^* | Pandoraea *^¥2,¥3^* | Roseburia | Shewanella |
| Myxococcus *^*1,*2,*3^* | Parabacteroides | Rothia | Shuttleworthia |
| Nannocystis *^*1,*2,*3^* | Paracoccus | Ruminococcus | Slackia *^θ1,θ3^* |
| Neisseria *^*2^* | Paraprevotella | Salmonella *^＄1,＄3^* | Sneathia |
| Nesterenkonia *^*2^* | Parvimonas | Sediminibacterium | Solitalea *^θ3^* |
| Nitrosovibrio *^*1,*2,*3^* | Pedobacter | Selenomonas | Sphingobium |
| Nitrospira | Peptococcus | Serratia | Sphingomonas |
| Novosphingobium | Peptoniphilus | Shewanella | Spirosoma *^θ2,θ3^* |
| Ochrobactrum | Peptostreptococcus | Shuttleworthia | Sporocytophaga *^θ1,θ3^* |
| Odoribacter | Phaeospirillum *^¥1,¥2,¥3^* | Simplicispira *^＄3^* | Sporosarcina *^θ2,θ3^* |
| Oscillospira | Phascolarctobacterium | Sneathia | Staphylococcus |
| Oxalobacter *^*2,*3^* | Phenylobacterium *^¥3^* | Solibacillus *^＄3^* | Stenotrophomonas |
| Paenibacillus *^*3^* | Phyllobacterium | Solirubrobacter *^＄1,＄3^* | Steroidobacter *^θ2^* |
| Paenisporosarcina *^*1,*2^* | Pilimelia *^¥1,¥3^* | Sphingobacterium *^＄3^* | Streptococcus |
| Paludibacter *^*3^* | Pimelobacter *^¥3^* | Sphingobium | Streptomyces |
| Pandoraea *^*2,*3^* | Planomicrobium | Sphingomonas | Succinivibrio |
| Parabacteroides | Plesiocystis | Staphylococcus | Sulfuricurvum |
| Paracoccus | Polaromonas *^¥3^* | Stenotrophomonas | Sulfuritalea *^θ1,θ2,θ3^* |
| Paraprevotella | Polynucleobacter *^¥3^* | Steroidobacter | Sutterella |
| Parasegitibacter *^*1,*2,*3^* | Pontibacter *^¥3^* | Streptacidiphilus *^＄1,＄3^* | Symbiobacterium *^θ2,θ3^* |
| Parvimonas | Porphyromonas | Streptococcus | Thermomonas *^θ3^* |
| Paucibacter *^*1,*2,*3^* | Prevotella | Streptomyces | Thermus |
| Pedobacter *^*1^* | Propionibacterium | Succinivibrio | Trabulsiella *^θ3^* |
| Pedomicrobium | Propionicimonas *^¥2,¥3^* | Sulfuricurvum | Treponema |
| Peptococcus | Propionivibrio *^¥1,¥2,¥3^* | Sutterella | Truepera *^θ3^* |
| Peptoniphilus | Prosthecobacter *^¥2^* | Tepidimonas *^＄3^* | Ureaplasma |
| Peptostreptococcus | Proteus | Thauera *^＄3^* | Varibaculum |
| Perlucidibaca *^*1,*2,*3^* | Pseudoclavibacter *^¥1,¥2,¥3^* | Thermus | Veillonella |
| Phascolarctobacterium | Pseudomonas | Thiobacillus *^＄3^* | Vogesella |
| Phenylobacterium *^*3^* | Pseudonocardia *^¥3^* | Thiovirga *^＄1,＄3^* | Wautersiella *^θ2,θ3^* |
| Phormidium *^*1,*3^* | Pseudoramibacter_Eubacterium *^¥1,¥3^* | Treponema | Williamsia *^θ1,θ3^* |
| Phyllobacterium | Pseudoxanthomonas | Turicibacter *^＄3^* | Zoogloea *^θ1^* |
| Pimelobacter *^*3^* | Psychrilyobacter *^¥1,¥2,¥3^* | Ureaplasma |  |
| Planktothrix *^*1,*3^* | Psychrobacter *^¥2,¥3^* | Vagococcus *^＄1,＄2,＄3^* |  |
| Planomicrobium | Pyramidobacter | Varibaculum |  |
| Pleomorphomonas *^*1,*2,*3^* | Ralstonia *^¥2^* | Veillonella |  |
| Plesiocystis | Ramlibacter | Vibrio *^＄3^* |  |
| Polaromonas *^*3^* | Rheinheimera *^¥2,¥3^* | Vogesella |  |
| Polynucleobacter *^*3^* | Rhodanobacter *^¥2,¥3^* | Zoogloea *^＄1^* |  |
| Pontibacter *^*3^* | Rhodobacter *^¥3^* |  |  |
| Porphyromonas | Rhodococcus |  |  |
| Prevotella | Rhodoferax *^¥3^* |  |  |
| Promicromonospora *^*1^* | Rhodoplanes |  |  |
| Propionibacterium | Rikenella *^¥1,¥3^* |  |  |
| Propionicimonas *^*2,*3^* | Roseburia |  |  |
| Prosthecobacter *^*2^* | Roseococcus *^¥1,¥2,¥3^* |  |  |
| Proteus | Roseomonas *^¥2^* |  |  |
| Pseudoalteromonas *^*1,*2,*3^* | Rothia |  |  |
| Pseudomonas | Rubellimicrobium *^¥2^* |  |  |
| Pseudonocardia *^*3^* | Rubrobacter *^¥2,¥3^* |  |  |
| Pseudoxanthomonas | Ruminococcus |  |  |
| Psychrobacter *^*2,*3^* | Saccharibacillus *^¥1,¥2,¥3^* |  |  |
| Pyramidobacter | Salinicoccus *^¥1,¥2,¥3^* |  |  |
| Ralstonia *^*2^* | Sediminibacterium |  |  |
| Ramlibacter | Selenomonas |  |  |
| Rheinheimera *^*2,*3^* | Serratia |  |  |
| Rhizobium *^*1,*2,*3^* | Shewanella |  |  |
| Rhodanobacter *^*2,*3^* | Shinella *^¥2,¥3^* |  |  |
| Rhodobacter *^*3^* | Shuttleworthia |  |  |
| Rhodococcus | Simplicispira *^¥3^* |  |  |
| Rhodoferax *^*3^* | Slackia *^¥1,¥2,¥3^* |  |  |
| Rhodoplanes | Sneathia |  |  |
| Roseateles *^*1,*2^* | Solibacillus *^¥3^* |  |  |
| Roseburia | Solitalea *^¥2^* |  |  |
| Roseomonas *^*2^* | Sphingobacterium *^¥3^* |  |  |
| Rothia | Sphingobium |  |  |
| Rubellimicrobium *^*2^* | Sphingomonas |  |  |
| Rubrivivax *^*2,*3^* | Sphingopyxis *^¥2,¥3^* |  |  |
| Rubrobacter *^*2,*3^* | Sporocytophaga *^¥1,¥2,¥3^* |  |  |
| Ruminococcus | Staphylococcus |  |  |
| Runella *^*1,*2,*3^* | Stenotrophomonas |  |  |
| Sediminibacterium | Streptococcus |  |  |
| Selenomonas | Streptomyces |  |  |
| Serratia | Succinivibrio |  |  |
| Shewanella | Sulfuricurvum |  |  |
| Shinella *^*2,*3^* | Sulfurimonas *^¥1,¥2,¥3^* |  |  |
| Shuttleworthia | Sutterella |  |  |
| Simplicispira *^*3^* | Tepidimonas *^¥3^* |  |  |
| Skermanella *^*1,*2,*3^* | Thauera *^¥3^* |  |  |
| Sneathia | Thermomonas *^¥2^* |  |  |
| Solibacillus *^*3^* | Thermus |  |  |
| Solitalea *^*2^* | Thiobacillus *^¥3^* |  |  |
| Sphingobacterium *^*3^* | Trabulsiella *^¥2^* |  |  |
| Sphingobium | Treponema |  |  |
| Sphingomonas | Trichococcus *^¥1,¥2,¥3^* |  |  |
| Sphingopyxis *^*2,*3^* | Truepera *^¥2^* |  |  |
| Sporosarcina *^*1,*2^* | Trueperella *^¥1,¥2,¥3^* |  |  |
| Staphylococcus | Turicibacter *^¥3^* |  |  |
| Stenotrophomonas | Ureaplasma |  |  |
| Steroidobacter *^*1^* | Varibaculum |  |  |
| Streptococcus | Veillonella |  |  |
| Streptomyces | Vibrio *^¥3^* |  |  |
| Succinivibrio | Virgisporangium *^¥1,¥2,¥3^* |  |  |
| Sulfuricurvum | Vogesella |  |  |
| Sutterella | Williamsia *^¥1,¥2,¥3^* |  |  |
| Symbiobacterium *^*1^* | Zoogloea *^¥1,¥3^* |  |  |
| Tepidibacter *^*1,*3^* |  |  |  |
| Tepidimonas *^*3^* |  |  |  |
| Thauera *^*3^* |  |  |  |
| Thermomonas *^*2^* |  |  |  |
| Thermus |  |  |  |
| Thiobacillus *^*3^* |  |  |  |
| Tissierella Soehngenia *^*1,*2,*3^* |  |  |  |
| Trabulsiella *^*2^* |  |  |  |
| Treponema |  |  |  |
| Truepera *^*2^* |  |  |  |
| Turicibacter *^*3^* |  |  |  |
| Turneriella *^*1,*2,*3^* |  |  |  |
| Ureaplasma |  |  |  |
| Varibaculum |  |  |  |
| Variovorax *^*1,*2,*3^* |  |  |  |
| Veillonella |  |  |  |
| Vibrio *^*3^* |  |  |  |
| Virgibacillus *^*1,*2,*3^* |  |  |  |
| Vogesella |  |  |  |
| Wautersiella *^*1,*2^* |  |  |  |
| Weissella *^*1,*2,*3^* |  |  |  |

*^a^* DM: diabetes mellitus; HLP: hyperlipidemia; HT: hypertension;

*^b *1^,^*2^*, and *^*3^*indicate that the genus was detected in the DM cohort but not in the DM+HT, DM+HLP, and DM+HT+HLP cohorts, respectively; *^¥1^*, *^¥2^*, and *^¥3^*indicate that the genus was detected in the DM+HT cohort but not in the DM, DM+HLP, and DM+HT+HLP cohorts, respectively;*^＄1^*,*^＄2^*, and *^＄3^* indicate that the genus was detected in the DM+HLP cohort but not in the DM, DM+HT, and DM+HT+HLP cohorts, respectively; ^θ1^,*^θ2^*, and *^θ3^*indicate that the genus was detected in the DM+HT+HLP cohort but not in the DM, DM+HT, and DM+HLP cohorts, respectively.
