# Supplementary material for: Alterations of Urinary Microbiota in Type 2 Diabetes Mellitus with Hypertension and/or Hyperlipidemia
Source: Front Physiol. 2017 Mar 3;8:126. doi: 10.3389/fphys.2017.00126 (PMC5334339; doi:10.3389/fphys.2017.00126)
Supplement: Supplementary file 2 [file Table2.DOC]

**TABLE S2 Relationships between fasting blood glucose and the relative abundance of bacteria at the genus level in the diabetes only cohort.**

| **Taxon** | **r-value** | ***p*-value** |
| --- | --- | --- |
| Atopobium | 0.40 | 0.045 |
| Cellvibrio | 0.43 | 0.035 |
| Rhodanobacter | 0.44 | 0.030 |
| Lampropedia | 0.64 | 0.001 |

A correlation analysis was carried out and the bacteria shown are those that were found to be correlated using a significance level of *p* < 0.05.
