# Supplementary material for: Alterations of Urinary Microbiota in Type 2 Diabetes Mellitus with Hypertension and/or Hyperlipidemia
Source: Front Physiol. 2017 Mar 3;8:126. doi: 10.3389/fphys.2017.00126 (PMC5334339; doi:10.3389/fphys.2017.00126)
Supplement: Supplementary file 3 [file Table3.DOC]

**TABLE S3 Relationships between blood pressure and the relative abundance of bacteria at the genus level in the diabetes plus hypertension** cohort

| **Systolic pressure** | | |  | **Diastolic pressure** | | |
| --- | --- | --- | --- | --- | --- | --- |
| **Taxon** | **r-value** | ***p*-value** |  | **Taxon** | **r-value** | ***p*-value** |
| Phascolarctobacterium | 0.42 | 0.039 |  | Acidovorax | -0.45 | 0.029 |
| Blausia | 0.59 | 0.003 |  | Allobaculum | -0.50 | 0.014 |
| Coprococcus | 0.50 | 0.014 |  | Ammooniphilus | -0.55 | 0.006 |
| Collinsella | 0.63 | 0.001 |  | Dermacoccus | -0.46 | 0.023 |
| Bilophila | 0.52 | 0.009 |  | Eggerthella | -0.51 | 0.011 |
| Lachnobacterium | -0.43 | 0.036 |  | Janthinobacterium | -0.56 | 0.004 |
| Anaeromyxobacter | -0.43 | 0.035 |  | Ochrobactrum | -0.44 | 0.033 |
| Adlercreutzia | 0.65 | 0.001 |  | Paludibacter | -0.50 | 0.013 |
| Cetobacterium | -0.41 | 0.047 |  | Psychrilyobacter | -0.50 | 0.014 |
| Geobacter | -0.41 | 0.047 |  | Rubellimicrobium | -0.61 | 0.002 |
| Mitsuokella | 0.49 | 0.014 |  | Serratia | -0.52 | 0.010 |
| Desulfobacca | -0.41 | 0.047 |  | Simplicispira | -0.51 | 0.011 |
| Catenibacterium | -0.41 | 0.047 |  | Succinivibrio | -0.52 | 0.009 |
| Methyloversatilis | -0.43 | 0.035 |  | Truepera | -0.50 | 0.014 |
| Geodermatophilus | -0.41 | 0.047 |  | Vibrio | -0.50 | 0.014 |
| Knoellia | -0.41 | 0.047 |  |  |  |  |
| Oxalobacter | -0.41 | 0.047 |  |  |  |  |
| Phascolarctobacterium | 0.42 | 0.039 |  |  |  |  |
| Pilimelia | -0.41 | 0.047 |  |  |  |  |
| Propionivibrio | -0.41 | 0.047 |  |  |  |  |
| Prosthecobacter | -0.41 | 0.047 |  |  |  |  |
| Sphingopyxis | -0.41 | 0.047 |  |  |  |  |

A correlation analysis was carried out and the bacteria shown are those that were found to be correlated using a significance level of *p* < 0.05.
