# Supplementary material for: Alterations of Urinary Microbiota in Type 2 Diabetes Mellitus with Hypertension and/or Hyperlipidemia
Source: Front Physiol. 2017 Mar 3;8:126. doi: 10.3389/fphys.2017.00126 (PMC5334339; doi:10.3389/fphys.2017.00126)
Supplement: Supplementary file 4 [file Table4.DOC]

**TABLE S4 Relationships between blood lipids and the relative abundance of bacteria at the genus level in the diabetes plus hyperlipidemia cohort.**

| **Triglyceride (mmol/L)** | | |  | **LDL-C (mmol/L) *^a^*** | | |  | **HDL-C (mmol/L) *^a^*** | | |  | **Total cholesterol (mmol/L)** | | |
| --- | --- | --- | --- | --- | --- | --- | --- | --- | --- | --- | --- | --- | --- | --- |
| **Taxon** | **r-value** | ***p*-value *^b^*** |  | **Taxon** | **r-value** | ***p*-value** |  | **Taxon** | **r-value** | ***p*-value** |  | **Taxon** | **r-value** | ***p*-value** |
| Faecalibacterium | 0.79 | 0.034 |  | Pseudomonas | -0.79 | 0.035 |  | Bifidobacterium | 0.80 | 0.030 |  | Pseudomonas | -0.77 | 0.043 |
| Blautia | 0.76 | 0.049 |  | Klebsiella | -0.77 | 0.045 |  | Ruminococcus | 0.77 | 0.041 |  | Veillonella | -0.78 | 0.039 |
| Aggregatibacter | 0.83 | 0.021 |  | Vogesella | -0.93 | 0.002 |  | Lachnospira | 0.80 | 0.033 |  | Halomonas | -0.77 | 0.042 |
| Bdellovibrio | 0.77 | 0.042 |  | Staphylococcus | -0.91 | 0.005 |  | Jonquetella | 0.83 | 0.022 |  | Chryseobacterium | -0.86 | 0.014 |
| Lachnobacterium | 0.83 | 0.021 |  | Stenotrophomonas | -0.85 | 0.017 |  | Pyramidobacter | 0.83 | 0.022 |  | Vogesella | -0.83 | 0.022 |
| Acidaminococcus | 0.83 | 0.021 |  | Agrobacterium | -0.77 | 0.043 |  | Kaistobacter | 0.83 | 0.022 |  | Staphylococcus | -0.77 | 0.043 |
| Succinivibrio | 0.83 | 0.021 |  | Brevibacterium | -0.92 | 0.004 |  | Desulfovibrio | 0.82 | 0.023 |  | Stenotrophomonas | -0.77 | 0.042 |
| Rikenella | 0.95 | 0.001 |  | Simplicispira | -0.92 | 0.004 |  | Moryella | 0.83 | 0.022 |  | Nitrospira | -0.81 | 0.028 |
| Epulopiscium | 0.83 | 0.021 |  |  |  |  |  | Butyricimonas | 0.81 | 0.026 |  | Paracoccus | -0.91 | 0.005 |
| Leptotrichia | 0.83 | 0.021 |  |  |  |  |  | Rhodoplanes | 0.83 | 0.022 |  | Brevibacterium | -0.96 | 0.001 |
| Eggerthella | 0.87 | 0.012 |  |  |  |  |  | Rhodococcus | 0.83 | 0.022 |  |  |  |  |
| Succinibibrio | 0.83 | 0.021 |  |  |  |  |  | Vibrio | 0.83 | 0.022 |  |  |  |  |
| Planomicrobium | 0.83 | 0.021 |  |  |  |  |  | PHenylobacterium | 0.83 | 0.022 |  |  |  |  |
| Anaerotruncus | 0.91 | 0.004 |  |  |  |  |  | Rhobacter | 0.83 | 0.022 |  |  |  |  |
| Actinobacillus | 0.83 | 0.021 |  |  |  |  |  | Turicibacter | 0.83 | 0.022 |  |  |  |  |
|  |  |  |  |  |  |  |  | Arenimonas | 0.83 | 0.022 |  |  |  |  |
|  |  |  |  |  |  |  |  | Peptococcus | 0.83 | 0.022 |  |  |  |  |
|  |  |  |  |  |  |  |  | Janthinobacterium | 0.83 | 0.022 |  |  |  |  |
|  |  |  |  |  |  |  |  | Treponema | 0.76 | 0.050 |  |  |  |  |
|  |  |  |  |  |  |  |  | Acidovorax | 0.83 | 0.022 |  |  |  |  |
|  |  |  |  |  |  |  |  | Lysinibacillus | 0.83 | 0.022 |  |  |  |  |
|  |  |  |  |  |  |  |  | Fluvicola | 0.83 | 0.022 |  |  |  |  |
|  |  |  |  |  |  |  |  | Arcobacter | 0.81 | 0.029 |  |  |  |  |
|  |  |  |  |  |  |  |  | Hyphomonas | 0.83 | 0.022 |  |  |  |  |
|  |  |  |  |  |  |  |  | Steroidobacter | 0.83 | 0.022 |  |  |  |  |
|  |  |  |  |  |  |  |  | Mycoplana | 0.83 | 0.022 |  |  |  |  |
|  |  |  |  |  |  |  |  | Azohydromonas | 0.83 | 0.022 |  |  |  |  |
|  |  |  |  |  |  |  |  | Eubacterium | 0.83 | 0.022 |  |  |  |  |
|  |  |  |  |  |  |  |  | Plesiocystis | 0.83 | 0.022 |  |  |  |  |

*^a^* LDL-C: low-density lipoprotein cholesterol; HDL-C: high-density lipoprotein cholesterol.

*^b^* A correlation analysis was carried out and the bacteria shown are those that were found to be correlated using a significance level of *p* < 0.05.
