# Supplementary material for: Alterations of Urinary Microbiota in Type 2 Diabetes Mellitus with Hypertension and/or Hyperlipidemia
Source: Front Physiol. 2017 Mar 3;8:126. doi: 10.3389/fphys.2017.00126 (PMC5334339; doi:10.3389/fphys.2017.00126)
Supplement: Supplementary file 5 [file Table5.DOC]

**TABLE S5 Relationships between blood pressure and blood lipids and the relative abundance of bacteria at the genus level in the diabetes plus hypertension and hyperlipidemia.**

| **Systolic pressure (mmHg)** | | |  | **Diastolic pressure (mmHg)** | | |  | **Triglyceride (mmol/L)** | | |  | **LDL-C (mmol/L) *^a^*** | | |  | **HDL-C (mmol/L) *^a^*** | | |  | **Total cholesterol (mmol/L)** | | |
| --- | --- | --- | --- | --- | --- | --- | --- | --- | --- | --- | --- | --- | --- | --- | --- | --- | --- | --- | --- | --- | --- | --- |
| Taxon | r-value | *p*-value *^b^* |  | Taxon | r-value | *p*-value |  | Taxon | r-value | *p*-value |  | Taxon | r-value | *p*-value |  | Taxon | r-value | *p*-value |  | Taxon | r-value | *p*-value |
| Serratia | 0.66 | 0.027 |  | Odoribacter | -0.63 | 0.039 |  | Prevotella | -0.67 | 0.023 |  | Bacteroides | 0.90 | 0.005 |  | Odoribacter | -0.66 | 0.028 |  | Ruminococcus | 0.63 | 0.031 |
| Sphingobium | 0.60 | 0.034 |  |  |  |  |  | Anaerococcus | -0.80 | 0.005 |  | Pseudomonas | 0.64 | 0.032 |  | Dermacoccus | 0.63 | 0.037 |  | Aeromonas | -0.63 | 0.031 |
| Succinivibrio | 0.66 | 0.027 |  |  |  |  |  | Serratia | -0.90 | 0.000 |  | Faecalibacterium | 0.79 | 0.004 |  |  |  |  |  | Stenotrophomonas | 0.63 | 0.038 |
| Acidovorax | 0.65 | 0.031 |  |  |  |  |  | Odoribacter | -0.67 | 0.023 |  | Phascolarctobacterium | 0.88 | 0.000 |  |  |  |  |  | Odoribacter | -0.64 | 0.035 |
| Pedobacter | 0.87 | 0.001 |  |  |  |  |  | Adlercreutzia | -0.90 | 0.000 |  | Megamonas | 0.78 | 0.005 |  |  |  |  |  | Actinobaculum | -0.70 | 0.017 |
| Willliamsia | 0.66 | 0.027 |  |  |  |  |  | Succinivibrio | -0.90 | 0.000 |  | Blautia | 0.87 | 0.000 |  |  |  |  |  | Butyricimonas | -0.70 | 0.017 |
| Mycoplana | 0.78 | 0.004 |  |  |  |  |  | Janthinobacterium | -0.90 | 0.000 |  | Coprococcus | 0.91 | 0.000 |  |  |  |  |  | Ureaplasma | 0.62 | 0.043 |
| Roseomonas | 0.66 | 0.027 |  |  |  |  |  | Pedobacter | -0.82 | 0.002 |  | Shewanella | 0.77 | 0.006 |  |  |  |  |  | Delftia | 0.64 | 0.034 |
| Curtobacterium | 0.66 | 0.027 |  |  |  |  |  | Williamsia | -0.90 | 0.000 |  | Parabacteroides | 0.77 | 0.006 |  |  |  |  |  | Brochothrix | -0.70 | 0.017 |
| Arenibacter | 0.66 | 0.027 |  |  |  |  |  | Roseomonas | -0.90 | 0.000 |  | Flavobacterium | 0.90 | 0.000 |  |  |  |  |  | Allobaculum | -0.70 | 0.017 |
| Sulfuritalea | 0.66 | 0.027 |  |  |  |  |  | Curtobacterium | -0.90 | 0.000 |  | Dorea | 0.82 | 0.002 |  |  |  |  |  | Providencia | -0.70 | 0.017 |
|  |  |  |  |  |  |  |  | Arenibacter | -0.90 | 0.000 |  | Vogesella | 0.83 | 0.001 |  |  |  |  |  | Cryocola | -0.70 | 0.017 |
|  |  |  |  |  |  |  |  | Sulfuritalea | -0.90 | 0.000 |  | Comamonas | 0.87 | 0.000 |  |  |  |  |  | Legionella | -0.70 | 0.017 |
|  |  |  |  |  |  |  |  |  |  |  |  | Roseburia | 0.85 | 0.001 |  |  |  |  |  | Wautersiella | 0.70 | 0.017 |
|  |  |  |  |  |  |  |  |  |  |  |  | Lysobacter | 0.87 | 0.001 |  |  |  |  |  | Promicromonospora | -0.70 | 0.017 |
|  |  |  |  |  |  |  |  |  |  |  |  | Paraprevotella | 0.87 | 0.001 |  |  |  |  |  |  |  |  |
|  |  |  |  |  |  |  |  |  |  |  |  | Kaistobacter | 0.88 | 0.000 |  |  |  |  |  |  |  |  |
|  |  |  |  |  |  |  |  |  |  |  |  | Collinsella | 0.80 | 0.003 |  |  |  |  |  |  |  |  |
|  |  |  |  |  |  |  |  |  |  |  |  | Mycoplasma | 0.85 | 0.001 |  |  |  |  |  |  |  |  |
|  |  |  |  |  |  |  |  |  |  |  |  | Candidatus | 0.85 | 0.001 |  |  |  |  |  |  |  |  |
|  |  |  |  |  |  |  |  |  |  |  |  | Agrobacterium | 0.85 | 0.001 |  |  |  |  |  |  |  |  |
|  |  |  |  |  |  |  |  |  |  |  |  | Ochrobactrum | 0.87 | 0.001 |  |  |  |  |  |  |  |  |
|  |  |  |  |  |  |  |  |  |  |  |  | Cupriavidus | 0.85 | 0.001 |  |  |  |  |  |  |  |  |
|  |  |  |  |  |  |  |  |  |  |  |  | Agromyces | 0.85 | 0.001 |  |  |  |  |  |  |  |  |
|  |  |  |  |  |  |  |  |  |  |  |  | Mycobacterium | 0.82 | 0.002 |  |  |  |  |  |  |  |  |
|  |  |  |  |  |  |  |  |  |  |  |  | Neisseria | 0.84 | 0.001 |  |  |  |  |  |  |  |  |
|  |  |  |  |  |  |  |  |  |  |  |  | Microbacterium | 0.70 | 0.017 |  |  |  |  |  |  |  |  |
|  |  |  |  |  |  |  |  |  |  |  |  | Brevibacterium | 0.68 | 0.022 |  |  |  |  |  |  |  |  |
|  |  |  |  |  |  |  |  |  |  |  |  | Azoarcus | 0.85 | 0.001 |  |  |  |  |  |  |  |  |
|  |  |  |  |  |  |  |  |  |  |  |  | Solitalea | 0.85 | 0.001 |  |  |  |  |  |  |  |  |
|  |  |  |  |  |  |  |  |  |  |  |  | Luteimonas | 0.85 | 0.001 |  |  |  |  |  |  |  |  |
|  |  |  |  |  |  |  |  |  |  |  |  | Fluviicola | 0.85 | 0.001 |  |  |  |  |  |  |  |  |
|  |  |  |  |  |  |  |  |  |  |  |  | Asticcacaulis | 0.85 | 0.001 |  |  |  |  |  |  |  |  |
|  |  |  |  |  |  |  |  |  |  |  |  | Helicobacter | 0.85 | 0.001 |  |  |  |  |  |  |  |  |
|  |  |  |  |  |  |  |  |  |  |  |  | Truepera | 0.85 | 0.001 |  |  |  |  |  |  |  |  |
|  |  |  |  |  |  |  |  |  |  |  |  | Inquilinus | 0.85 | 0.001 |  |  |  |  |  |  |  |  |
|  |  |  |  |  |  |  |  |  |  |  |  | Azospirillum | 0.85 | 0.001 |  |  |  |  |  |  |  |  |

*^a^* LDL-C: low-density lipoprotein cholesterol; HDL-C: high-density lipoprotein cholesterol.

*^b^* A correlation analysis was carried out and the bacteria shown are those that were found to be correlated using a significance level of *p* < 0.05.
