# Supplementary material for: Alterations of Urinary Microbiota in Type 2 Diabetes Mellitus with Hypertension and/or Hyperlipidemia
Source: Front Physiol. 2017 Mar 3;8:126. doi: 10.3389/fphys.2017.00126 (PMC5334339; doi:10.3389/fphys.2017.00126)
Supplement: Supplementary file 6 [file Table6.DOC]

**TALBE S6 Nutrient and medicine intake of the DM, DM+HT, DM+HLP, and DM+HT+HLP cohorts *^a^.***

| Parameters *^b,c^* | DM (n = 25) | DM+HT (n = 24) | DM+HLP (n = 7) | DM+HT+HLP (n = 11) |
| --- | --- | --- | --- | --- |
| Nutrients |  | | | |
| Energy (Kcal/d) | 1483.39±601.54*^*^* | 1090.61±255.38*^*＄^* | 1388.13±473.39*^＄^* | 1090.09±445.76 |
| Protein (g/d) | 68.19±29.77*^*^* | 51.88±20.50*^*＄^* | 74.02±32.35*^＄^* | 53.67±26.93 |
| Fat (g/d) | 68.59±35.90*^*^* | 47.79±19.26*^*^* | 68.06±37.38 | 46.81±22.91 |
| Carbohydrate (g/d) | 170.40±72.38*^*^* | 132.32±24.46*^*^* | 138.65±27.63 | 142.67±59.77 |
| Vitamin A (μg/d) | 789.11±479.16 | 586.39±566.18 | 695.64±373.65 | 636.37±932.68 |
| Vitamin B1 (mg/d) | 3.76±7.29*^¥^* | 6.78±6.08*^＄^* | 1.93±3.36*^＄θ^* | 11.94±10.82*^¥θ^* |
| Vitamin B2 (mg/d) | 28.77±60.29*^¥^* | 56.50±52.19*^＄^* | 15.65±39.07*^＄θ^* | 82.87±67.11*^¥θ^* |
| Vitamin B5 (mg/d) | 23.02±23.78 | 23.07±20.29 | 13.25±4.57 | 29.38±24.65 |
| Vitamin C (mg/d) | 87.52±56.98*^*¥^* | 43.08±62.62*^*^* | 85.84±44.50*^θ^* | 31.72±42.62*^¥θ^* |
| Vitamin E (mg/d) | 277.14±511.22*^¥^* | 523.46±381.14*^＄^* | 131.53±305.66*^＄θ^* | 664.94±482.61*^¥θ^* |
| Calcium (mg/d) | 1010.66±741.39 | 1123.30±531.50 | 993.84±467.66 | 1334.36±802.05 |
| Phosphorus (mg/d) | 1333.11±743.731 | 1815.11±1216.06 | 1353.13±609.83 | 2259.00±1434.54 |
| Potassium (mg/d) | 1718.71±1124.42*^*¥^* | 940.82±972.12*^*^* | 1561.07±858.07 | 720.69±701.14*^¥^* |
| Sodium (mg/d) | 2304.25±2447.43*^¥^* | 1106.23±1823.80*^＄^* | 2892.24±1844.91*^＄θ^* | 493.56±1033.61*^¥θ^* |
| Magnesium (mg/d) | 230.81±176.22*^*¥^* | 88.48±141.28*^*＄^* | 283.75±161.12*^＄θ^* | 40.53±71.79*^¥θ^* |
| Iron (mg/d) | 27.24±16.156 | 24.62±12.98 | 31.09±13.54 | 32.12±27.25 |
| Znic (mg/d) | 9.63±6.35 | 6.27±7.12 | 10.18±5.26 | 5.24±6.08 |
| Selenium (mg/d) | 31.97±20.76*^*¥^* | 15.55±22.74*^*＄^* | 42.65±22.55*^＄θ^* | 6.47±7.21*^¥θ^* |
| Copper (mg/d) | 13.06±26.53 | 22.22±21.42*^＄^* | 4.91±5.96*^＄θ^* | 27.70±26.41*^θ^* |
| Manganese (mg/d) | 15.60±25.24 | 24.99±19.64*^＄^* | 7.45±6.56*^＄θ^* | 32.69±24.55*^θ^* |
| Saturated fatty acid (g/d) | 14.45±9.48 | 12.28±5.09 | 13.12±6.87 | 12.96±6.92 |
| Monounsaturated fatty acid (g/d) | 21.67±14.56*^*^* | 14.11±8.59*^*^* | 21.65±14.44 | 14.90±7.63 |
| Polyunsaturated fatty acid (g/d) | 19.69±12.81 | 13.56±8.15 | 19.69±13.71 | 13.97±7.80 |
| Water (ml/d) | 2395.28 ± 655.96 | 2595.92 ± 952.86 | 2546.47 ± 662.43 | 2263.49 ± 774.09 |
| Medicine (%) | | | | |
| Aspirin | 2 (8.00%) *^¥^* | 5 (26.32%) | 2 (28.57%) | 6 (54.54) *^¥^* |
| Gingseng | 5 (20.00%) | 2 (8.33%) | 0 (0.00%) | 1 (9.09%) |
| Dendrobii | 2 (8.00%) | 2 (8.33%) | 0/7 (0.00%) | 3 (27.27%) |

*^a^* DM: diabetes mellitus; HLP: hyperlipidemia; HT: hypertension; N/A: not applicable.

*^b^* Independent *t*-test and Pearson’s chi-square test were used to test for significant differences (*p* < 0.05) in each variable between the four cohorts.

*^c^* Nutrient and water intake were assessed using the Chinese Food Frequency Questionnaire which includes supplement intake.

*^*^* represents a significant difference between the DM and DM+HT cohorts; *^¥^* represents a significant difference between the DM and DM+HT+HLP cohorts; *^＄^* represents significant difference between the DM+HT and DM+HLP cohorts; and *^θ^* represents a significant difference between the DM+HLP and DM+HT+HLP cohorts.
