# Supplementary material for: Alterations of Urinary Microbiota in Type 2 Diabetes Mellitus with Hypertension and/or Hyperlipidemia
Source: Front Physiol. 2017 Mar 3;8:126. doi: 10.3389/fphys.2017.00126 (PMC5334339; doi:10.3389/fphys.2017.00126)
Supplement: Supplementary file 7 [file Table7.doc]

**TABLE S7 Relationships between nutrient intake and the relative abundance of bacteria at the genus level**

| **Nutrients** | **Taxon** | **r-value** | ***p*-value** |
| --- | --- | --- | --- |
| Energy (Kcal/d) | | | |
|  | Pseudomonas | 0.310 | 0.011 |
|  | Flavobacterium | 0.264 | 0.032 |
|  | Deinococcus | 0.311 | 0.011 |
|  | Akkermansia | 0.282 | 0.022 |
|  | Brevundimonas | 0.360 | 0.003 |
|  | Flavisolibacter | 0.276 | 0.025 |
|  | Microbacterium | 0.284 | 0.021 |
|  | Paracoccus | 0.270 | 0.028 |
|  | Pseudoxanthomonas | 0.270 | 0.028 |
|  | Thermononas | 0.313 | 0.010 |
|  | Arenimonas | 0.346 | 0.004 |
|  | Arthrobacter | 0.247 | 0.046 |
|  | Janthinobacterium | 0.245 | 0.047 |
|  | Dehalobacterium | 0.250 | 0.043 |
|  | Dpulopiscium | 0.350 | 0.004 |
|  | Skermanella | 0.278 | 0.024 |
|  | Runella | 0.278 | 0.024 |
|  | Aminobacter | 0.274 | 0.026 |
|  | Sphingopyxis | 0.261 | 0.035 |
|  | Caldilinea | 0.301 | 0.014 |
|  | Rubrivivax | 0.278 | 0.024 |
|  | Alcanivorax | 0.270 | 0.028 |
|  | Haliscomenobacter | 0.278 | 0.024 |
|  | Paucibacter | 0.278 | 0.024 |
|  | Rhodanobacter | 0.268 | 0.030 |
|  | Variovorax | 0.259 | 0.035 |
|  | Pontibacter | 0.268 | 0.029 |
|  | Arsenicicoccus | 0.313 | 0.011 |
|  | Nitrosovibrio | 0.278 | 0.024 |
|  | Azospira | 0.278 | 0.024 |
|  | Actinotalea | 0.247 | 0.045 |
|  | Tepidibacter | 0.278 | 0.024 |
|  | Tissierella Soehngenia | 0.406 | 0.001 |
|  | Pseudoalteromonas | 0.278 | 0.024 |
|  | Lacibacter | 0.278 | 0.024 |
|  | Chitinophaga | 0.278 | 0.024 |
|  | Emticicia | 0.278 | 0.024 |
|  | Ardenscatena | 0.278 | 0.024 |
|  | Pleomorphomonas | 0.301 | 0.014 |
|  | Parasegtibacter | 0.278 | 0.024 |
| Protein (g/d) | | | |
|  | Pseudomonas | 0.336 | 0.006 |
|  | Halomonas | 0.286 | 0.020 |
|  | Akkermansia | 0.272 | 0.027 |
|  | Microbacerium | 0.412 | 0.001 |
|  | Paracoccus | 0.280 | 0.023 |
|  | Mitsuokella | 0.275 | 0.025 |
|  | Arthrobacter | 0.334 | 0.006 |
|  | Epulopiscium | 0.287 | 0.020 |
|  | Pseudonocardia | 0.286 | 0.020 |
|  | Roseateles | 0.250 | 0.043 |
|  | Actinobacillus | 0.259 | 0.036 |
|  | Caulobacter | 0.265 | 0.032 |
|  | Pontibacter | 0.349 | 0.004 |
|  | Simplicispira | 0.252 | 0.041 |
|  | Bradyrhizobium | 0.250 | 0.043 |
|  | Actinotalea | 0.332 | 0.006 |
|  | Tissierella Soehngenia | 0.336 | 0.006 |
| Fat (g/d) | | | |
|  | Pseudomonas | 0.264 | 0.033 |
|  | Megasphaera | 0.276 | 0.025 |
|  | Akkermansia | 0.413 | 0.001 |
|  | Mitsuokella | 0.404 | 0.001 |
|  | Arthrobacter | 0.267 | 0.030 |
|  | Rothia | 0.310 | 0.011 |
|  | Epulopiscium | 0.424 | 0.000 |
|  | Pseudonocardia | 0.265 | 0.031 |
|  | Akkermansia | 0.413 | 0.001 |
|  | Mitsuokella | 0.404 | 0.001 |
|  | Arthrobacter | 0.267 | 0.030 |
|  | Rothia | 0.310 | 0.011 |
|  | Epulopiscium | 0.424 | 0.000 |
|  | Tissierella Soehngenia | 0.487 | 0.000 |
| Carbohydrate (g/d) | | | |
|  | Pseudomonas | 0.260 | 0.035 |
|  | Flavobacterium | 0.426 | 0.000 |
|  | Vogesella | 0.381 | 0.002 |
|  | Deinococcus | 0.474 | 0.000 |
|  | Lysobacter | 0.386 | 0.001 |
|  | Kaistobacter | 0.417 | 0.000 |
|  | Bdellovibrio | 0.335 | 0.006 |
|  | Ochrobactrum | 0.350 | 0.004 |
|  | Cupriavidus | 0.344 | 0.005 |
|  | Ramlibacter | 0.269 | 0.029 |
|  | Rhodoplanes | 0.353 | 0.004 |
|  | Flavihumibacter | 0.269 | 0.029 |
|  | Gemmatimonas | 0.285 | 0.020 |
|  | Brevundimonas | 0.573 | 0.000 |
|  | Flavisolibacter | 0.466 | 0.000 |
|  | Hydrogenophaga | 0.373 | 0.002 |
|  | Paracoccus | 0.319 | 0.009 |
|  | Pseudoxanthomonas | 0.403 | 0.001 |
|  | Dechloromonas | 0.380 | 0.002 |
|  | Azoarcus | 0.272 | 0.027 |
|  | Thermononas | 0.436 | 0.000 |
|  | Arenimonas | 0.553 | 0.000 |
|  | Thauera | 0.275 | 0.026 |
|  | Methylibium | 0.314 | 0.010 |
|  | Hymenobacter | 0.372 | 0.002 |
|  | Janthinobacterium | 0.421 | 0.000 |
|  | Solitalea | 0.392 | 0.001 |
|  | Adhaeribacter | 0.431 | 0.000 |
|  | Steroidobacter | 0.413 | 0.001 |
|  | Perlucidibaca | 0.469 | 0.000 |
|  | Skermanella | 0.443 | 0.000 |
|  | Runella | 0.440 | 0.000 |
|  | Pedomicrobium | 0.326 | 0.008 |
|  | Polaromonas | 0.372 | 0.002 |
|  | Mycoplana | 0.355 | 0.003 |
|  | Plesiocystis | 0.316 | 0.010 |
|  | Aminobacter | 0.431 | 0.000 |
|  | Aquicella | 0.442 | 0.000 |
|  | Sphingopyxis | 0.428 | 0.000 |
|  | Calilinea | 0.487 | 0.000 |
|  | Rubrivivax | 0.440 | 0.000 |
|  | Alcanivorax | 0.422 | 0.000 |
|  | Haliscomenobacter | 0.440 | 0.000 |
|  | Paucibacter | 0.440 | 0.000 |
|  | Rhodanobacter | 0.273 | 0.027 |
|  | Afifella | 0.440 | 0.000 |
|  | Chthoniobacter | 0.440 | 0.000 |
|  | Limnohabitans | 0.343 | 0.005 |
|  | Nannocystis | 0.440 | 0.000 |
|  | Catellatospora | 0.440 | 0.000 |
|  | Variovorax | 0.421 | 0.000 |
|  | Curtobacterium | 0.258 | 0.036 |
|  | Arsenicicoccus | 0.494 | 0.000 |
|  | Nitrosovibrio | 0.440 | 0.000 |
|  | Azospira | 0.440 | 0.000 |
|  | Tepidibacter | 0.440 | 0.000 |
|  | Knoellia | 0.342 | 0.005 |
|  | Myxococcus | 0.440 | 0.000 |
|  | Pseudoalteromonas | 0.440 | 0.000 |
|  | Lacibacter | 0.440 | 0.000 |
|  | Chitinophaga | 0.440 | 0.000 |
|  | Emticicia | 0.440 | 0.000 |
|  | Ardenscatena | 0.440 | 0.000 |
|  | Pleomorphomonas | 0.485 | 0.000 |
|  | Parasegitibacter | 0.440 | 0.000 |
|  | Ensifer | 0.440 | 0.000 |
| Vitamin B1(mg/d) | | | |
|  | Peptoniphilus | 0.280 | 0.023 |
|  | Corynebacterium | 0.323 | 0.008 |
|  | Finegoldia | 0.347 | 0.004 |
|  | Haemophilus | 0.333 | 0.006 |
|  | Morganella | 0.412 | 0.001 |
|  | Kocuria | 0.374 | 0.002 |
|  | Dehalobacterium | 0.286 | 0.020 |
|  | Slenomonas | 0.261 | 0.035 |
|  | Slackia | 0.261 | 0.035 |
|  | Plesiocystis | 0.317 | 0.010 |
|  | Fervidobacterium | 0.436 | 0.000 |
|  | Corynebacterium | 0.323 | 0.008 |
|  | Finegoldia | 0.347 | 0.004 |
|  | Haemophilus | 0.333 | 0.006 |
|  | Microbacterium | 0.403 | 0.001 |
|  | Morganella | 0.412 | 0.001 |
|  | Kocuria | 0.374 | 0.002 |
|  | Peptococcus | 0.244 | 0.048 |
|  | Dehalobacterium | 0.286 | 0.020 |
|  | Slackia | 0.261 | 0.035 |
|  | Plesiocystis | 0.317 | 0.010 |
|  | Leucobacter | 0.413 | 0.001 |
|  | Marinibacillus | 0.249 | 0.044 |
| Vitamin B2 (mg/d) | | | |
|  | Peptoniphilus | 0.286 | 0.020 |
|  | Corynebacterium | 0.305 | 0.013 |
|  | Sediminibacterium | 0.268 | 0.030 |
|  | Finegoldia | 0.357 | 0.003 |
|  | Haemophilus | 0.284 | 0.021 |
|  | Microbacterium | 0.345 | 0.005 |
|  | Kocuria | 0.324 | 0.008 |
|  | Peptococcus | 0.261 | 0.034 |
|  | Dehalobacterium | 0.256 | 0.038 |
|  | Bosea | 0.264 | 0.032 |
| Vitamin C (mg/d) | | | |
|  | Peptoniphilus | -0.369 | 0.002 |
|  | Acinetobacter | 0.282 | 0.022 |
|  | Campylobacter | -0.317 | 0.010 |
|  | Clostridium | 0.355 | 0.003 |
|  | Finegoldia | -0.289 | 0.019 |
|  | Desulfovibrio | 0.377 | 0.002 |
|  | Mycoplasma | 0.286 | 0.020 |
|  | Novophingobium | 0.313 | 0.010 |
|  | Devosia | 0.404 | 0.001 |
|  | Methyloversatilis | 0.246 | 0.047 |
|  | Pimelobacter | 0.272 | 0.027 |
| Vitamin E (mg/d) | | | |
|  | Peptoniphilus | 0.344 | 0.005 |
|  | Campylobacter | 0.274 | 0.026 |
|  | Shuttleworthia | -0.243 | 0.049 |
|  | Facklamia | 0.306 | 0.012 |
| Potassium (mg/d) | | | |
|  | Peptoniphilus | -0.249 | 0.044 |
|  | Corynebacterium | -0.302 | 0.014 |
|  | Finegoldia | -0.314 | 0.010 |
|  | Deinococcus | 0.255 | 0.039 |
|  | Kocuria | -0.243 | 0.049 |
|  | Actinobacillus | 0.292 | 0.017 |
|  | Arsenicicoccus | 0.246 | 0.046 |
| Sodium (mg/d) | | | |
|  | Peptoniphilus | -0.299 | 0.015 |
|  | Campylobacter | -0.253 | 0.040 |
|  | Anaerococcus | -0.244 | 0.048 |
|  | Sutterella | 0.267 | 0.030 |
|  | Finegoldia | -0.246 | 0.046 |
|  | Akkermansia | 0.516 | 0.000 |
|  | Candidatus | 0.401 | 0.001 |
|  | Misuokella | 0.591 | 0.000 |
|  | Rothia | 0.482 | 0.000 |
| Magnesium (mg/d) | | | |
|  | Prevotella | -0.254 | 0.039 |
|  | Pseudomonas | 0.279 | 0.023 |
|  | Peptoniphilus | -0.329 | 0.007 |
|  | Acinetobacter | 0.349 | 0.004 |
|  | Corybacterium | -0.254 | 0.039 |
|  | Campylobacter | -0.292 | 0.017 |
|  | Finegoldia | -0.293 | 0.017 |
|  | Nitrospira | 0.302 | 0.014 |
|  | Flavisolibacter | 0.332 | 0.006 |
|  | Brevibacterium | 0.254 | 0.039 |
|  | Thermomonas | 0.283 | 0.021 |
|  | Arthrobacter | 0.246 | 0.047 |
|  | Novosphingobium | 0.352 | 0.004 |
|  | Adhaeribacter | 0.283 | 0.021 |
| Selenium (mg/d) | | | |
|  | Pseudomonas | 0.302 | 0.014 |
|  | Peptoniphilus | -0.320 | 0.009 |
|  | Campylobacter | -0.287 | 0.020 |
|  | Oscillospira | 0.252 | 0.041 |
|  | Finegoldia | -0.264 | 0.032 |
|  | Akkermansia | 0.348 | 0.004 |
|  | Candidatus | 0.352 | 0.004 |
|  | Ochrobactrum | 0.307 | 0.012 |
|  | Nitrospira | 0.364 | 0.003 |
|  | Flavisolibacter | 0.255 | 0.039 |
|  | Cetobacterium | 0.339 | 0.005 |
|  | Delftia | 0.283 | 0.021 |
|  | Geobacter | 0.333 | 0.006 |
|  | Thermonas | 0.315 | 0.010 |
|  | Thauera | 0.295 | 0.016 |
|  | Mitsuokella | 0.349 | 0.004 |
|  | Arthrobacter | 0.251 | 0.042 |
|  | Rothia | 0.248 | 0.045 |
|  | Novosphingobium | 0.296 | 0.016 |
|  | Epulopiscium | 0.292 | 0.017 |
|  | Desulfobacca | 0.351 | 0.004 |
|  | Catenibacterium | 0.339 | 0.005 |
|  | Methyloversatilis | 0.246 | 0.046 |
|  | Desulfococcus | 0.315 | 0.010 |
|  | Prosthecobacter | 0.359 | 0.003 |
|  | Pilimelia | 0.339 | 0.005 |
|  | Sphingopyxis | 0.285 | 0.020 |
|  | Cellulomonas | 0.254 | 0.040 |
|  | Pontibacter | 0.310 | 0.011 |
|  | Simplicispira | 0.285 | 0.020 |
| Copper (mg/d) | | | |
|  | Corynebacterium | 0.305 | 0.013 |
|  | Megasphaera | 0.286 | 0.020 |
|  | Helcococcus | 0.299 | 0.015 |
|  | Finegoldia | 0.273 | 0.027 |
|  | Haemophilus | 0.306 | 0.013 |
|  | Facklamia | 0.333 | 0.006 |
|  | Lachnobacterium | 0.255 | 0.039 |
|  | Microbacterium | 0.429 | 0.000 |
|  | Abiotrophia | 0.274 | 0.026 |
|  | Dehalobacterium | 0.306 | 0.013 |
|  | Kaistia | 0.286 | 0.020 |
| Monounsaturated fatty acid (g/d) | | | |
|  | Pseudomonas | 0.246 | 0.047 |
|  | Peptoniphilus | -0.262 | 0.034 |
|  | Akkermansia | 0.492 | 0.000 |
|  | Misuokella | 0.489 | 0.000 |
|  | Arthrobacter | 0.249 | 0.043 |
|  | Rothia | 0.450 | 0.000 |
|  | Epulopiscium | 0.534 | 0.000 |
|  | Pseudonocardia | 0.257 | 0.038 |
|  | Pontibacter | 0.265 | 0.032 |
|  | Bradyrhizobium | 0.263 | 0.033 |
|  | Actinotalea | 0.263 | 0.033 |

A correlation analysis was carried out and the bacteria shown are those that were found to be correlated using a significance level of *p* < 0.05
